# Bystander activated CD8^+^ T cells mediate neuropathology during viral infection via antigen-independent cytotoxicity

**DOI:** 10.1038/s41467-023-44667-0

**Published:** 2024-02-05

**Authors:** Elizabeth Balint, Emily Feng, Elizabeth C. Giles, Tyrah M. Ritchie, Alexander S. Qian, Fatemeh Vahedi, Amelia Montemarano, Ana L. Portillo, Jonathan K. Monteiro, Bernardo L. Trigatti, Ali A. Ashkar

**Affiliations:** 1 McMaster Immunology Research Centre, Department of Medicine, McMaster University, Hamilton, ON Canada; 2 Thrombosis and Atherosclerosis Research Institute, Department of Biochemistry and Biomedical Sciences, McMaster University, Hamilton Health Sciences, Hamilton, ON Canada

**Keywords:** Viral infection, Neuroimmunology, Cytotoxic T cells, Innate immunity

## Abstract

Although many viral infections are linked to the development of neurological disorders, the mechanism governing virus-induced neuropathology remains poorly understood, particularly when the virus is not directly neuropathic. Using a mouse model of Zika virus (ZIKV) infection, we found that the severity of neurological disease did not correlate with brain ZIKV titers, but rather with infiltration of bystander activated NKG2D^+^CD8^+^ T cells. Antibody depletion of CD8 or blockade of NKG2D prevented ZIKV-associated paralysis, suggesting that CD8^+^ T cells induce neurological disease independent of TCR signaling. Furthermore, spleen and brain CD8^+^ T cells exhibited antigen-independent cytotoxicity that correlated with NKG2D expression. Finally, viral infection and inflammation in the brain was necessary but not sufficient to induce neurological damage. We demonstrate that CD8^+^ T cells mediate virus-induced neuropathology via antigen-independent, NKG2D-mediated cytotoxicity, which may serve as a therapeutic target for treatment of virus-induced neurological disease.

## Abstract (web summary)

Many viral infections are linked to the development of neurological disorders. Here, Balint et al use a mouse model of Zika virus infection to show that it is immune cells (NKG2D+CD8+ T cells) that cause infection-associated paralysis, rather than the virus itself.

## Introduction

Many viral infections have been linked to development of debilitating neurological disease that is difficult to treat, with severe cases resulting in long-term disability or death. Although viral infection has long been associated with neurological disease, the mechanism through which viruses cause neuropathology remains controversial. While the prevailing hypothesis suggests that a high viral load results in more severe damage and cell death, some viruses show little to no infection of the central nervous system (CNS) and the viral load cannot explain the extent of neurological damage. For example, influenza, Ebola, dengue, and measles virus infections have been associated with various neurological sequelae, with limited evidence of CNS infection or uncontrolled replication^1–5^. Furthermore, it is possible that other viruses can induce similar clinical manifestations^2^. In recent years, Zika virus (ZIKV) and SARS-CoV-2 infections have also been linked to neurological sequelae, including encephalitis and Guillain Barre Syndrome (GBS), but the underlying mechanism of brain damage remains unknown^6–9^. New evidence recently identified Epstein-Barr virus (EBV) as a causal agent for the development of Multiple Sclerosis (MS), while only a small proportion of patients exhibit EBV infection in the CNS^10^. Furthermore, while direct viral infection and uncontrolled replication may contribute to apoptosis of infected neurons, astrocytes, and other cells of the CNS, the complex role of the immune system in neurological disease in the presence and absence of CNS viral infection remains poorly understood. Thus, we sought to clarify the role of viral replication and the immune response in virus-induced neurological disease.

To investigate mechanisms of virus-induced neuropathology, we used a Zika virus (ZIKV) infection model in *Ifnar*^*-/-*^ mice. ZIKV is an emerging mosquito-borne and sexually transmitted virus that is associated with the development of microcephaly in growing fetuses and GBS in adults^8,11–14^. In this mouse model of ZIKV infection, ZIKV invades the CNS via infected monocytes and subsequently infects several parts of the spinal cord and brain, including the motor cortex and hippocampus^15–21^. While ZIKV mouse models are known to induce severe neuropathology, most commonly resulting in hindlimb paralysis that resembles GBS, the underlying mechanism of this disease remains poorly understood. While some groups suggest that increased viral replication in the CNS is responsible for development of neurological disease^22–25^, others suggest the damage is immune-mediated. Jurado et al. (2018) reported that ZIKV-induced paralysis is mediated by CD8^+^ T cells following increased blood-brain-barrier permeability^18^. However, the antigen specificity of these cells has not been examined, and it remains unclear what shifts the balance from a beneficial antiviral T cell response to an uncontrolled T cell-mediated disease.

CD8^+^ T cells are well-established as adaptive immune cells with antigen specificity, but recent studies of liver disease indicate that this may be an oversimplification. Innate-like CD8^+^ T cells have been identified as key mediators of disease in studies of hepatitis A, C, D, and E virus infections, as well as non-alcoholic steatohepatitis^26–30^. These studies demonstrate that inflammatory cytokines can promote ‘bystander activation’ of CD8^+^ T cells in the absence of TCR signaling, resulting in expression of a Natural Killer (NK) cell activation receptor, NKG2D. NKG2D ligands are typically expressed on stressed cells, including cancer and virally infected cells, but it has been suggested that some epithelial or endothelial cells may constitutively express these ligands or upregulate expression in response to cytokine stimulation^31–35^. Similarly, Kim et al. (2018) observed that both hepatitis A virus-infected cells and uninfected cells from acute hepatitis A patients express NKG2D ligands, indicating that bystander activated T cells may mediate tissue damage by excessive killing of stressed cells, independent of viral infection^26^. Despite previous investigation of bystander activated CD8^+^ T cells in the liver, it is unclear if this phenomenon occurs in an immune-privileged environment, such as the CNS, and whether these T cells can contribute to virus-induced neurological diseases.

In this study, we find that the viral load does not correlate with severity of disease. Instead, we provide evidence that ZIKV-induced brain damage is mediated by an uncontrolled CD8^+^ T cell response. Furthermore, we identify antigen-independent cytotoxicity of bystander activated CD8^+^ T cells as a critical mediator of virus-induced neurological disease. This mechanism of virus-induced neuropathology is a promising avenue for investigation of treatments to prevent and treat debilitating neurological disease.

## Results

### ZIKV-infected *Ifnar*^*-/-*^ mice exhibit clinical symptoms of neuropathology associated with immune cell infiltration of the brain

**Fig. 1:**
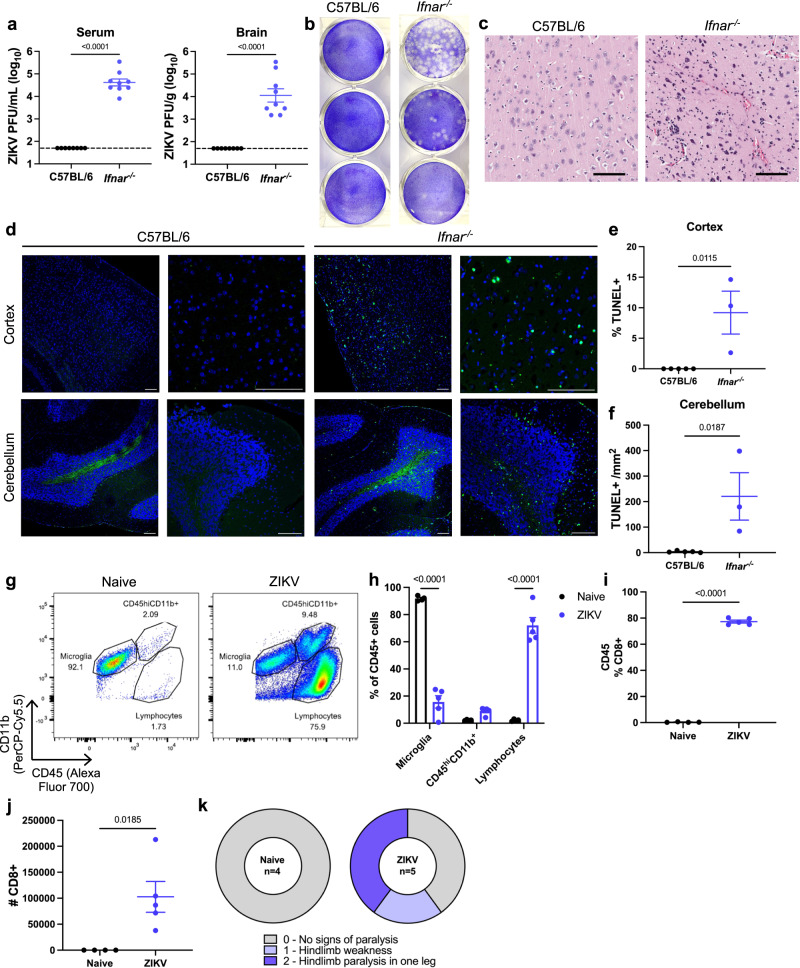
ZIKV-infected *Ifnar*^*-/-*^ mice exhibit clinical symptoms of neuropathology associated with immune cell infiltration of the brain. C57BL/6 and *Ifnar*^*-/-*^ mice were infected with 4 × 10^5^ PFU ZIKV via FTPD. **a** Serum was collected at 3 dpi and brains were isolated at 7 dpi to quantify ZIKV by plaque assay (*n* =5, repeated once with similar results). **b** Representative images of a ZIKV plaque assay of serially diluted serum samples. **c** Representative images of H&E staining of the cortex of brains isolated from C57BL/6 and *Ifnar*^*-/-*^ mice. Scale bar represents 100 μm. **d** Representative images of TUNEL staining of sagittal sections of cerebral cortex and cerebellum at ×10 and ×40 magnification. Scale bar represents 100 μm. **e** Quantification of % TUNEL^+^ nuclei in the cortex and **f** the # of TUNEL^+^ cells /mm^2^ within the granular layer (*n* =5, 3). **g** Representative flow cytometry plots of CD45^+^ cells isolated from PBS-treated (naïve) and ZIKV-infected *Ifnar*^*-/-*^ mice at 7 dpi. See also Supplementary Fig. 1 for gating strategy. **h** Quantification of brain immune cell populations following PBS or ZIKV infection, represented as the percentage of CD45^+^ cells (*n* =4, 5). **i** Comparison of CD8^+^ T cells as a percentage of CD45^+^ cells and **j** absolute count of CD8^+^ T cells in the brains of PBS and ZIKV-infected mice (*n* =4, 5). **k** ZIKV-infected *Ifnar*^*-/-*^ mice were assessed at 7 dpi for clinical symptoms of paralysis (*n* =5). Data represent mean ± SEM (**a**, **e**, **f**, **h**, **i**, **j**). Statistical significance was determined by two-tailed t-test (**a**, **e**, **f**, **i**, **j**) or two-way ANOVA with Sidak’s multiple comparison test (**h**). See also Supplementary Fig. 1. Source data are provided as a Source Data file.

It is well known that ZIKV possesses mechanisms to evade type I IFN signaling in humans^36,37^. To model ZIKV in mice, several groups have employed the use of mice deficient in type I IFN signaling. Likewise, we find that *Ifnar*^*-/-*^ mice are susceptible to ZIKV infection, identified by high titers of infectious ZIKV particles in the serum and brains of ZIKV-infected mice at 3- and 7-days post-infection (dpi), respectively (Fig. 1a, b). Histological comparison of brains of ZIKV-infected C57BL/6 and *Ifnar*^*-/-*^ mice exhibited lymphocyte infiltration into the brains of infected *Ifnar*^*-/-*^ mice, while C57BL/6 mice were unaffected (Fig. 1c). Using terminal deoxynucleotidyl transferase dUTP nick end labeling (TUNEL), we identified significant cell death in the brains of ZIKV-infected *Ifnar*^*-/-*^ mice in the cerebral cortex and the granular layer of the cerebellum (Fig. 1d–f). Cell death in these regions have previously been correlated with neurological disease in ZIKV and Tacaribe virus infection^18,38^. Further characterization of the immune cell populations in naïve and ZIKV-infected *Ifnar*^−/−^ mouse brains using flow cytometry demonstrated a significant influx of lymphocytes, mainly CD8^+^ T cells, at 7 dpi (Fig. 1g–j and Supplementary Fig. 1). At the culmination of high viral infection, cell death, and immune cell infiltration in the brain at 7-8 dpi, a proportion of ZIKV-infected mice exhibited clinical symptoms of neurological disease, including hindlimb weakness or paralysis (Fig. 1k).

### Innate immune activation via IL-12/18 treatment of *Ifnar*^*-/-*^ mice reduces ZIKV infection but enhances ZIKV-induced neuropathology

**Fig. 2:**
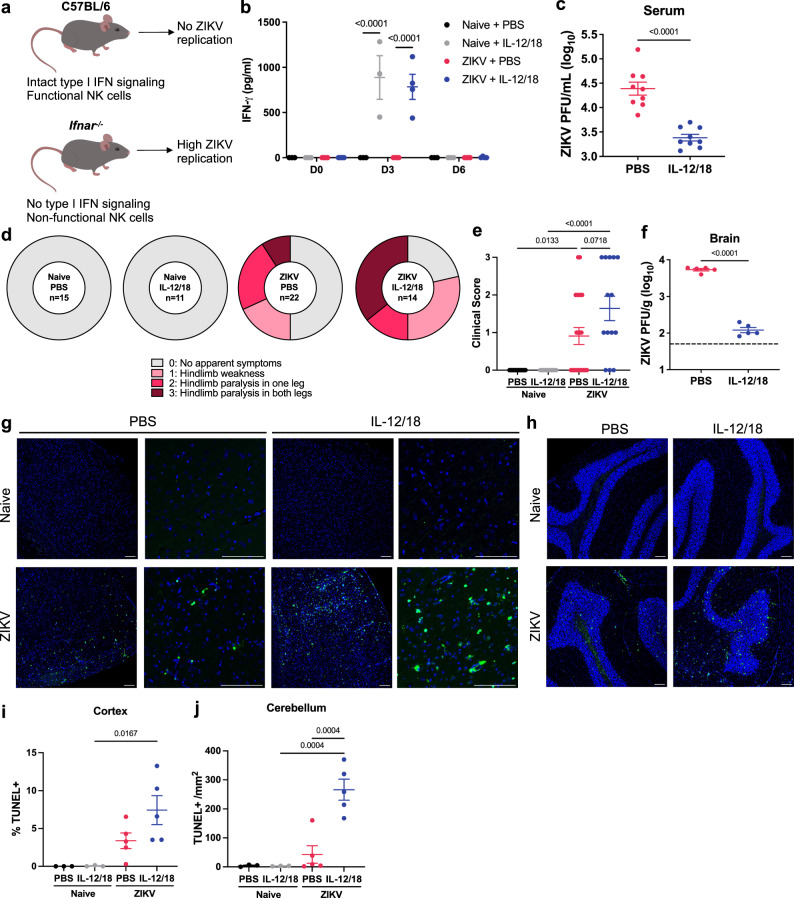
Innate immune activation via IL-12/18 treatment of *Ifnar*^*-/-*^ mice reduces ZIKV infection but enhances ZIKV-induced neuropathology. **a** Visual representation of differences between C57BL/6 and *Ifnar*^−/−^ mice and their susceptibility to ZIKV infection. **b** *Ifnar*^−/−^ mice were infected with PBS or 4x10^5^ ZIKV PFU via FTPD and IP injected with PBS or IL-12/18 at 1 and 2 dpi. Serum was collected at 3 dpi and assessed for IFN-γ production (*n* =3, 3, 4, 4, repeated three times with similar results) or **c** viremia by plaque assay (*n* =9). **d** At 7 dpi, mice were assessed for clinical symptoms of paralysis (*n* =15, 11, 22, 14). **e** Quantification of **d** (*n* =15, 11, 22, 14). **f** ZIKV-infected mice were administered PBS or IL-12/18 at 0-3 dpi and brains were collected at 7 dpi for detection of infectious ZIKV particles (*n* =5). Dashed lines represent limit of detection. **g** Representative images of TUNEL staining of the cerebral cortex or **h** cerebellum. Scale bar represents 100 μm. **i** Quantification of % TUNEL^+^ nuclei in the cortex or **j** # of TUNEL^+^ nuclei/mm^2^ of the granular layer. Data represent mean ± SEM (**b**, **c**, **e**, **f**, **i**, **j**) of two (**i**) or three independent experiments (**d**, **e**). Statistical significance was determined by repeated measures (**b**) or ordinary (**e**) two-way ANOVA with Tukey’s multiple comparisons test and two-tailed Student’s *t* test (**c**, **f**, **i**, **j**). See also Supplementary Fig. 2. Source data are provided as a Source Data file.

Although *Ifnar*^*-/-*^ mice are frequently used to model ZIKV infection in humans, these mice possess limitations in innate immunity that renders them more susceptible to severe disease. Due to their deficiencies in type I IFN signaling, NK cell maturation, activation, and IFN-γ production is limited in *Ifnar*^*-/-*^ mice (Fig. 2a)^39–41^. Since NK cells play a critical role in controlling early viral replication, their dysfunction can result in uncontrolled viral replication, which has been suggested to facilitate severe neurological disease. To investigate the role of viral replication in neuropathology, we administered the cytokines IL-12 and IL-18 (IL-12/18) at 1 and 2 dpi to ZIKV-infected *Ifnar*^*-/-*^ mice, which are known to exert a synergistic effect on NK cell activation and IFN-γ production^42,43^. This treatment rescued NK cell IFN-γ production, as demonstrated by increased levels of IFN-γ in the serum and detection of intracellular IFN-γ in NK1.1^+^ cells at 3 dpi (Fig. 2b and Supplementary Fig. 2a–e). As expected, IL-12/18 treatment also significantly reduced serum ZIKV titers at 3 dpi (Fig. 2c and Supplementary Fig. 2f) and remained low at 5 dpi (Supplementary Fig. 2g). Surprisingly, despite the enhanced protection against ZIKV, these mice exhibited increased incidence and severity of symptoms of paralysis (Fig. 2d, e). Similarly, mice administered IL-12/18 at 0-3 dpi demonstrated a significant decrease in brain ZIKV titers, despite worse symptoms of paralysis (Fig. 2f). Consistent with clinical outcomes, we found that ZIKV-infected IL-12/18-treated mice exhibited increased cell death in the cerebral cortex and cerebellum compared to ZIKV-infected mice given control PBS injections (Fig. 2g–j). Together, these results suggest that the viral load is not directly correlated with severity of neuropathology.

### Increased CD8^+^ T cell response in the brain correlates with exacerbated neurological disease in ZIKV-infected mice

**Fig. 3:**
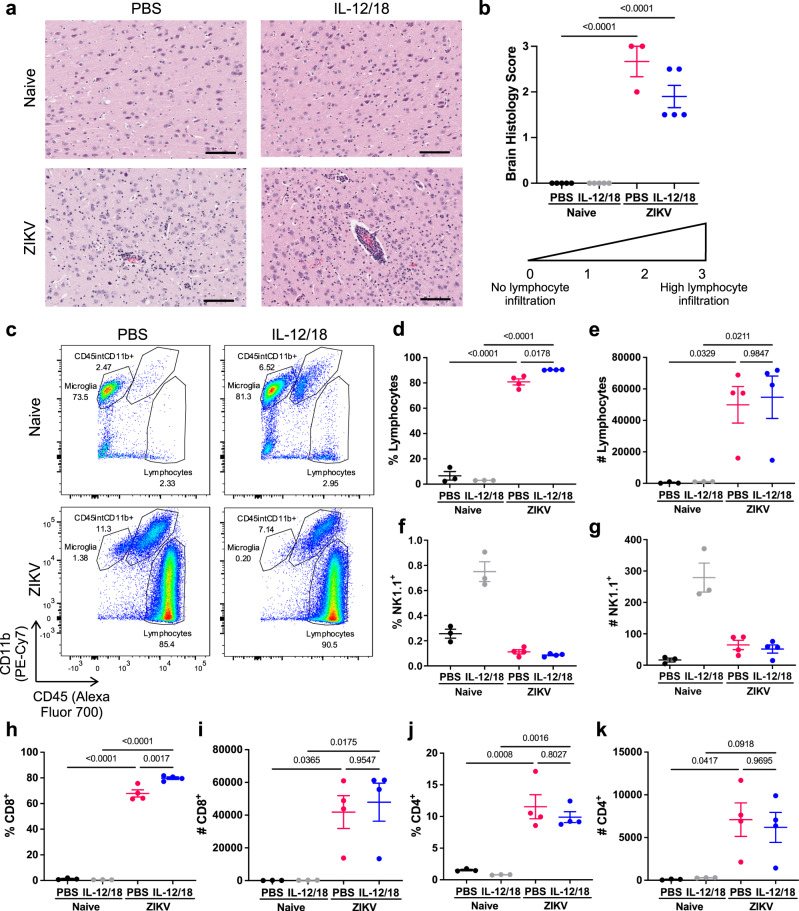
Increased CD8^+^ T cell response in the brain correlates with exacerbated neurological disease in ZIKV-infected mice. *Ifnar*^*-/-*^ mice were infected with 4x10^5^ PFU via FTPD and administered PBS or IL-12/18 at 0-3 dpi (**a**, **b**) or 1 and 2 dpi (**c**–**k**). **a** Representative images of H&E staining of the cortex of brains from naïve and ZIKV-infected mice treated with PBS or IL-12/18. Scale bar represents 100 μm. **b** Quantification of **a**. Data represent average scores per brain of two blinded measurements on a scale of 0–3, with 0 being no lymphocyte infiltration and 3 being high levels of infiltration and perivascular cuffing. **c** Representative flow cytometry plots of CD45^+^ cells isolated from brains of naïve and ZIKV-infected mice at 7 dpi. **d**–**k** CD45^+^ cells were analyzed for proportion or total number of **d**, **e** lymphocytes, **f**, **g** NK1.1^+^, **h**, **i** CD8^+^, and **j**, **k** CD4^+^ (CD8^-^) cells (*n* =3, 3, 4, 4). Data represents mean ± SEM (**b**, **d**–**k**). Statistical significance was determined by two-way ANOVA with Tukey’s multiple comparisons test (**b**, **d**, **e**, **h**–**k**). See also Supplementary Fig. 1. Source data are provided as a Source Data file.

As IL-12/18 are inflammatory cytokines capable of activating innate and adaptive immune cells, we hypothesized that IL-12/18 treatment increases pathology by promoting immune-mediated brain damage. H&E staining of brains demonstrated prominent leukocyte infiltration and perivascular cuffing in ZIKV-infected mice and those administered IL-12/18 (Fig. 3a, b). As seen previously, CD8^+^ T cells comprise the majority of infiltrating immune cells in the brains of ZIKV-infected mice (Fig. 1f). Furthermore, a previous study suggested that CD8^+^ T cells may contribute to ZIKV-induced neuropathology^18^. Thus, we sought to determine if IL-12/18 treatment altered T cell and NK cell infiltration in the brain, as IL-12/18 can activate both of these populations^42–45^. As expected, ZIKV-infected mice demonstrated a significant increase in lymphocytes (Fig. 3c–e). Although NK cells in the periphery appeared to be activated due to IFN-γ production at 3 dpi (Fig. 2a and Supplementary Fig. 2a–e), few NK cells infiltrated the brains of ZIKV-infected mice, suggesting that these cells are unlikely to contribute to immune cell-mediated brain damage (Fig. 3f, g). Instead, mice treated with IL-12/18 demonstrated a significant increase in the frequency of lymphocytes, and specifically CD8^+^ T cells, in the brain while absolute counts of these populations were not significantly altered (Fig. 3d, e, h, i). Furthermore, frequency and counts of CD4^+^ T cells were not altered by IL-12/18 treatment (Fig. 3j, k). Overall, these data suggest that IL-12/18 increased ZIKV-induced neuropathology by enhancing a pathogenic CD8^+^ T cell response, despite lower viral titers in the brain.

### ZIKV infection induces a population of CD8^+^ T cells with a bystander activated phenotype

While CD8^+^ T cells have been suggested to contribute to ZIKV-induced paralysis, the mechanism behind this immunopathology remains unclear. Our observations of reduced systemic and brain ZIKV infection indicated that the T cells should have fewer targets for antigen-specific killing, yet these mice exhibited greater incidence and severity of paralysis. Previous research groups have suggested that inflammatory cytokines, such as IL-12/18, can promote ‘bystander activation’ of CD8^+^ T cells in the absence of TCR signaling, which could explain the exacerbated disease in IL-12/18-treated mice^26,44–47^. Thus, we hypothesized that CD8^+^ T cells undergo bystander activation and perform antigen-independent killing in the CNS of ZIKV-infected mice.

**Fig. 4:**
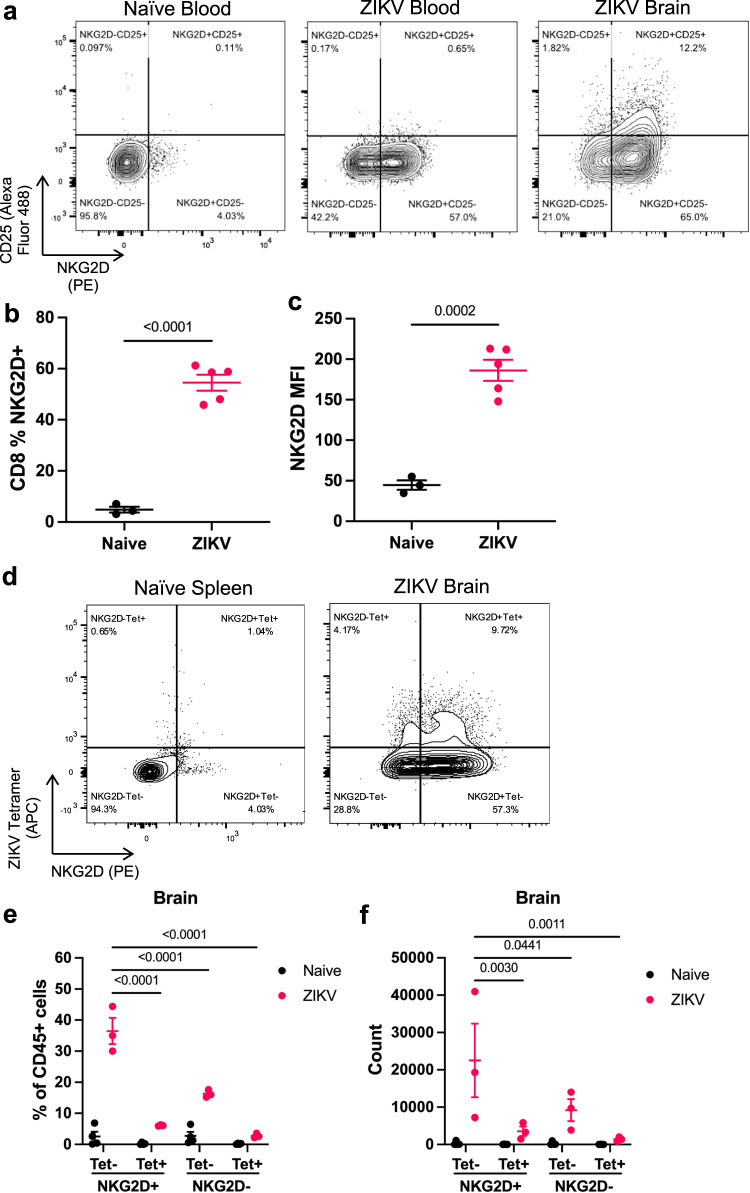
ZIKV infection induces a population of CD8^+^ T cells with a bystander activated phenotype. *Ifnar*^−/−^ mice were infected with PBS (naïve) or 4x10^5^ PFU ZIKV via FTPD. At 7 dpi, cells from the blood and brain were isolated for flow cytometry analysis. **a** Representative flow plots of NKG2D and CD25 staining of CD8^+^ T cells in the blood and brain of naïve and ZIKV-infected mice. **b** Proportion of blood CD8^+^ T cells that are NKG2D^+^(*n* =3, 5). **c** MFI of NKG2D on blood CD8^+^ T cells (*n* =3, 5). **d** Representative flow cytometry plots of APC-conjugated tetramer and NKG2D expression on CD8^+^ T cells in the naïve spleen and ZIKV-infected brains. **e** Proportion of CD45^+^ cells in naïve or ZIKV-infected brains that are CD8^+^ T cells and their expression of NKG2D and tetramer staining (*n* =3). **f** Counts of CD8^+^ T cells subset on NKG2D and tetramer staining (*n* =3). Data represent mean ± SEM (**b**, **c**, **e**, **f**) and represent one of two independent experiments with similar results. Statistical significance was determined by two-tailed t-test (**b**, **c**) and two-way ANOVA with Tukey’s multiple comparison test (**e**, **f**). Tet tetramer, MFI mean fluorescence intensity. See also Supplementary Fig. 3. Source data are provided as a Source Data file.

Bystander activated T cells have been demonstrated to express high levels of the NK cell receptor NKG2D and perform NKG2D-mediated cytotoxicity^26,47^. These cells also differ from antigen-specific T cells due to their lack of CD25 (IL-2R_α_) expression, which is associated with TCR signaling^48–50^. Thus, we sought to determine if CD8^+^ T cells from ZIKV-infected mice exhibit characteristics of bystander activated T cells. To do so, we isolated immune cells from the brains of ZIKV-infected *Ifnar*^*-/-*^ mice at the onset of paralysis at 7 dpi. CD8^+^ T cells isolated from the blood and brains of ZIKV-infected mice demonstrated an increase in % NKG2D^+^ cells and mean NKG2D expression compared to CD8^+^ T cells isolated from the blood of naïve mice (Fig. 4a–c). Likewise, the majority of CD8^+^ T cells in the blood and brains of ZIKV-infected mice were NKG2D^+^CD25^-^ (Fig. 4a). Additional phenotypic analysis demonstrated that these are conventional, TCR-β^+^CD8^+^ T cells that also express high levels of CXCR6, as seen in recent studies of liver bystander activated T cells (Supplementary Fig. 3)^27,30^. In an independent experiment, we further confirmed that most brain-infiltrating CD8^+^ T cells were not specific for the immunodominant E protein epitope, as shown by flow cytometry detection of CD8^+^ T cells bound to an APC-conjugated ZIKV E_294-302_ tetramer (Fig. 4d–f)^51,52^. Instead, the majority of immune cells in ZIKV-infected mice exhibited a Tet^-^NKG2D^+^ phenotype resembling bystander activated T cells, indicating a potential role for bystander killing in the CNS (Fig. 4d–f).

### NKG2D blockade and CD8^+^ T cell depletion protect against ZIKV-induced neuropathology

**Fig. 5:**
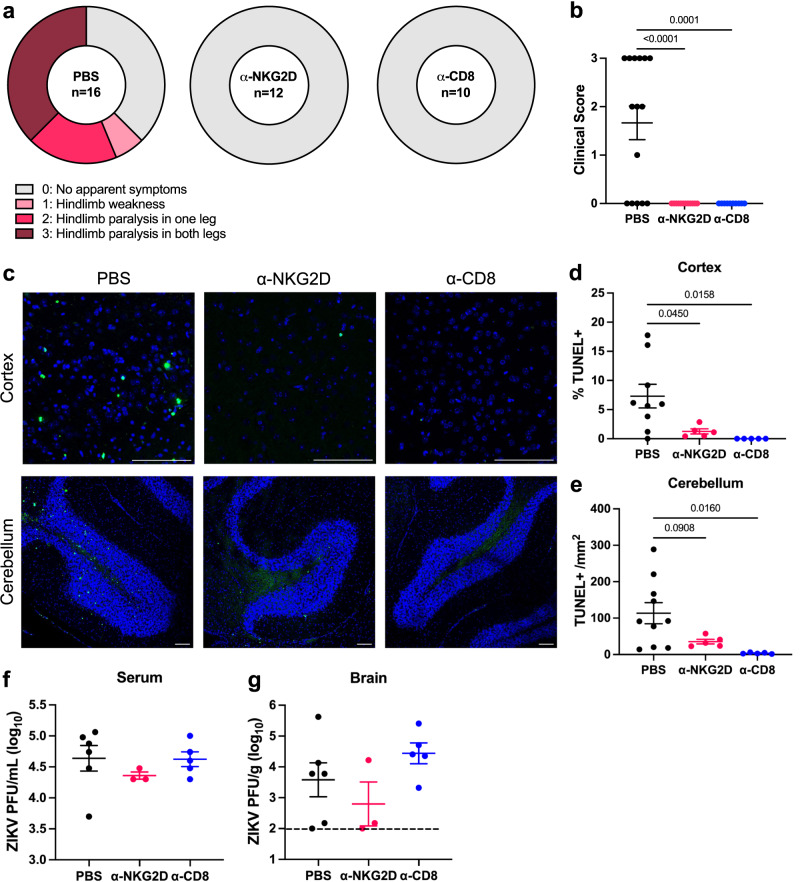
NKG2D blockade and CD8^+^ T cell depletion protect against ZIKV-induced neuropathology. *Ifnar*^*-/-*^ mice were infected with 4x10^5^ PFU via FTPD and treated with PBS, α-NKG2D, or α-CD8 antibody. **a** At 7 dpi, mice were assessed for clinical symptoms of paralysis (*n* =16, 12, 10; data represent three independent experiments). **b** Quantification of **a**. **c** Representative TUNEL staining of cerebral cortex and cerebellum at 7 dpi. Scale bar represents 100 μm. **d** Quantification of % TUNEL^+^ nuclei in the cortex or **e** # of TUNEL^+^ nuclei/mm^2^ of the granular layer of the cerebellum (*n* =9, 5, 5). **f** Serum was collected at 3 dpi and **g** brains were collected at 7 dpi for ZIKV quantification via plaque assay (*n* =5, 3, 5, data represent one of two independent experiments with similar results). Dashed lines represent limit of detection. Data represents mean ± SEM (**b**, **d**–**f**, **g**). Statistical significance was assessed by one-way ANOVA with Tukey’s multiple comparisons test (**b**, **d**–**f**, **g**, differences were not significant in **f**, **g**). See also Supplementary Fig. 4. Source data are provided as a Source Data file.

Following identification of cells with a bystander T cell phenotype, we investigated if blocking NKG2D or depleting CD8^+^ T cells would prevent antigen-independent killing and subsequently limit the development of paralysis. Depletion of CD8^+^ T cells or blockade of NKG2D on CD8^+^ T cells in the brain and spleen was confirmed by flow cytometry analysis (Supplementary Fig. 4a–g). Compared to PBS-treated controls, α-NKG2D and α-CD8 treated mice did not exhibit any symptoms of paralysis following ZIKV infection (Fig. 5a, b). Likewise, α-NKG2D and α-CD8 treatments significantly reduced cell death in the cortex and cerebellum compared to PBS-treated mice (Fig. 5c–e). This was not due to a difference in ZIKV titers and the cytopathic effects of viral replication, as we did not observe a significant difference in the serum at 3 dpi or in the brain at 7 dpi (Fig. 5f, g). These results further indicate that CD8^+^ T cells are necessary for ZIKV-induced neuropathology, which may occur through innate-like, NKG2D-mediated cytotoxicity.

### ZIKV infection in mice generates bystander activated CD8^+^ T cells capable of antigen-independent cytotoxicity

**Fig. 6:**
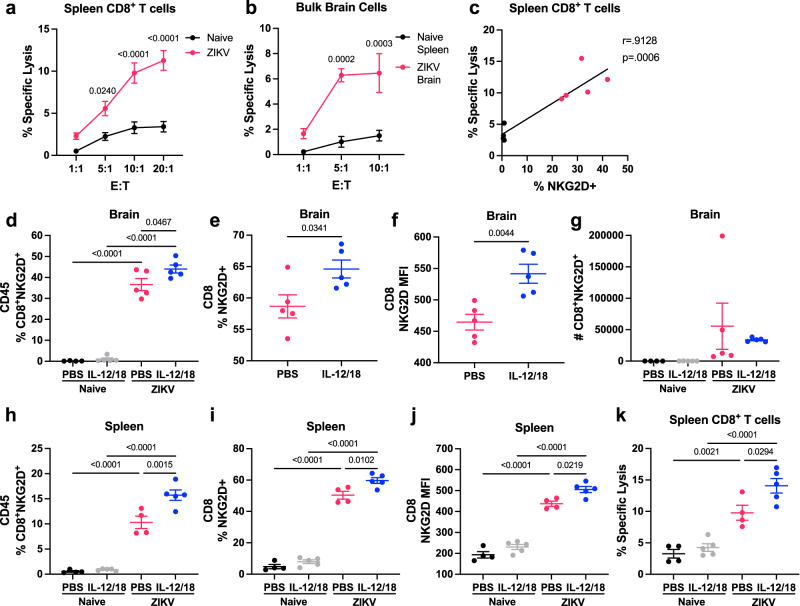
ZIKV infection in mice generates bystander activated CD8^+^ T cells capable of antigen-independent cytotoxicity. *Ifnar*^*-/-*^ mice were infected with PBS (naïve) or 4x10^5^ PFU ZIKV via FTPD. **a** At 7 dpi, spleens were harvested and CD8^+^ T cells isolated by negative selection. A cytotoxicity assay was performed by incubating CFSE-labeled YAC-1 cells with spleen CD8^+^ T cells at various effector:target (E:T) ratios (*n* =4, 5, repeated once with similar results). Each datapoint represents the mean of technical duplicates. **b** Bulk brain cells from ZIKV-infected mice were pooled, with 5 brains per sample (*n* =3) and compared to cytotoxicity of CD8^+^ T cells isolated from naïve mouse spleens (*n* =3, repeated once with similar results). Each datapoint represents the mean of technical duplicates. **c** Correlation of the proportion of spleen CD8^+^ T cells that were NKG2D^+^ with specific lysis at the 20:1 E:T ratio. **d**–**k** *Ifnar*^*-/-*^ mice were infected with PBS (naïve) or ZIKV and treated with PBS or IL-12/18 at 1 and 2 dpi. Brains were harvested for flow cytometry analysis and spleen CD8^+^ T cells were isolated for cytotoxicity assay at 7 dpi. **d** The proportion of brain CD45^+^ cells that were CD8^+^NKG2D^+^ (*n* =4, 5, 5, 5). **e** Proportion of brain CD8^+^ T cells from ZIKV-infected mice that were NKG2D^+^ (*n* =5). **f** MFI of NKG2D on brain CD8^+^ T cells from ZIKV-infected mice (*n* =5). **g** Count of CD8^+^NKG2D^+^ T cells in the brains of naïve and ZIKV-infected mice treated with PBS or IL-12/18 (*n* =4, 5, 5, 5). **h** Proportion of spleen CD45^+^ cells that were CD8^+^NKG2D^+^ (*n* =4, 5, 4, 5). **i** Proportion of spleen CD8^+^ T cells that were NKG2D^+^ (*n* =4, 5, 4, 5). **j** MFI of NKG2D on spleen CD8^+^ T cells (*n* =4, 5, 4, 5). **k** % specific lysis at 10:1 E:T ratios (*n* =4-5). Data represents mean ± SEM of one of two independent experiments with similar results (**a**, **b**, **d**–**k**). Statistical significance was determined by two-way ANOVA with Sidak’s multiple comparison test (**a, b**) or Tukey’s multiple comparison test (**d**, **g**–**k**), linear regression (**c**), and two-tailed Student’s *t* test (**e**, **f**). MFI mean fluorescence intensity. Source data are provided as a Source Data file.

While we identified T cells with a bystander phenotype, and blockade of NKG2D reduced cell death and neurological outcomes, the functional ability of these cells to perform nonspecific killing remained unknown. To determine if CD8^+^ T cells from ZIKV-infected mice were capable of antigen-independent cytotoxicity, we established a bystander cytotoxicity assay against YAC-1 tumor cells. YAC-1 cells are commonly used targets for mouse NK cell cytotoxicity assays and are known to express NKG2D ligands, but are typically resistant to T cell killing^53^. We first assessed antigen-independent killing using CD8^+^ T cells isolated at 7 dpi from the spleens of naïve or ZIKV-infected *Ifnar*^*-/-*^ mice. CD8^+^ T cells isolated from ZIKV-infected mice exhibited antigen-independent killing against tumor cells when compared to T cells isolated from naïve mice (Fig. 6a). Similarly, we found that pooled bulk brain cells from ZIKV-infected mice were also capable of killing YAC-1 tumor cells, indicating that this phenomenon with isolated spleen CD8^+^ T cells reliably represents bystander activated T cell function in the brain (Fig. 6b). Furthermore, at the 20:1 ratio, the % of spleen CD8^+^ T cells that were NKG2D^+^appeared to correlate with antigen-independent cytotoxicity (*r* = 0.9128 and *p* = 0.0006; Fig. 6c).

Since we previously observed increased severity of neurological disease in mice treated with IL-12/18, we compared brain CD8^+^ T cell NKG2D expression in both PBS and IL-12/18 treated ZIKV-infected mice (Supplementary Fig. 5a). ZIKV-infected mice treated with IL-12/18 exhibited a significant increase in the proportion of CD45^+^ immune cells that were NKG2D^+^CD8^+^ T cells, specifically due to an increase in Tet^-^NKG2D^+^CD8^+^ T cells, compared to PBS-treated ZIKV-infected mice (Fig. 6d and Supplementary Fig. 5b). IL-12/18 treatment also increased the proportion of CD8^+^ T cells that were NKG2D^+^ and enhanced NKG2D expression on the CD8^+^ T cells, suggesting that IL-12/18 signaling in vivo in the context of infection can enhance bystander T cell activation and function (Fig. 6e, f). Additionally, IL-12/18 treatment did not significantly alter the absolute count of NKG2D^+^CD8^+^ T cells in the brain, suggesting that IL-12/18 treatment promotes an imbalance in the CD8^+^ T cell response (Fig. 6g and Supplementary Fig. 5c). Similar to the brain, we found that IL-12/18 treatment increased frequency of CD8^+^NKG2D^+^ T cells, Tet^-^NKG2D^+^CD8^+^ T cells, and NKG2D expression on CD8^+^ T cells in the spleen of ZIKV-infected mice (Fig. 6h–j and Supplementary Fig. 5d). This increased NKG2D expression was associated with greater killing ability, as spleen CD8^+^ T cells from ZIKV-infected, IL-12/18-treated mice demonstrated significantly higher antigen-independent killing compared to ZIKV-infected mice treated with PBS (Fig. 6k). Altogether, these results indicate that CD8^+^ T cells from ZIKV-infected mice are capable of antigen-independent killing of YAC-1 tumor cells, and that this cytotoxicity is exacerbated by inflammatory stimuli, such as IL-12/18 treatment.

### ZIKV infection of α-IFNAR-treated C57BL/6 mice promotes bystander activation independent of viral load

**Fig. 7:**
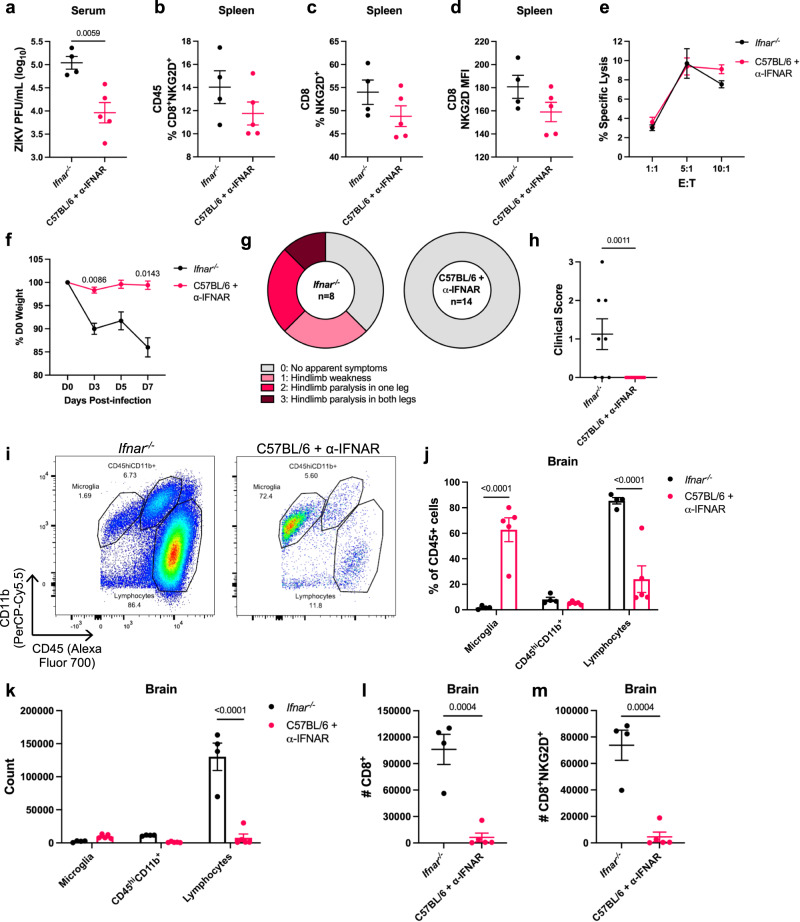
ZIKV infection of α-IFNAR-treated C57BL/6 mice promotes bystander activation independent of viral load. C57BL/6 mice were treated with α-IFNAR antibody at −1, 0, 1, 3, 5 dpi and infected via FTPD with 1.4 × 10^6^ ZIKV PFU. *Ifnar*^*-/-*^ mice were infected via FTPD with 4 × 10^5^ ZIKV PFU. **a** Viral titer in the serum at 3 dpi (*n* =4, 5). **b** Spleen cells were isolated for flow cytometry at 8 dpi and CD45^+^ cells were assessed for % CD8^+^NKG2D^+^ T cells (*n* =4, 5). **c** Spleen CD8^+^ T cells were assessed for %NKG2D^+^ or D) NKG2D MFI (*n* =4, 5). **e** At 8 dpi, spleen CD8^+^ T cells were isolated via negative selection. A cytotoxicity assay was performed by incubating CFSE-labeled YAC-1 cells with spleen CD8^+^ T cells at various effector:target (E:T) ratios (*n* =4, 5). Each datapoint represents the mean of technical duplicates. **f** Weight loss at a % of D0 starting weight (*n* =4, 5). **g** Mice were assessed for clinical symptoms of paralysis at 8 dpi (*n* =8, 14; data represent two independent experiments). **h** Quantification of clinical scores in **g** (*n* =8, 14). **i** Representative flow cytometry plots of brains of ZIKV-infected *Ifnar*^*-/-*^ and α-IFNAR-treated C57BL/6 mice. **j** Quantification of **i** (*n* =4, 5). **k** Counts of immune cell populations from **i** (*n* =4, 5). **l** Counts of CD8^+^ and **m** CD8^+^NKG2D^+^ in ZIKV-infected *Ifnar*^*-/-*^ and α-IFNAR-treated C57BL/6 mice (*n* =4, 5). Data represents mean ± SEM (**a**–**f**, **h**, **j**–**m**) of one of two independent experiments with similar results. Statistical significance was determined by two-tailed Student’s *t* test (**a**–**d**, **h**, **l**, **m**) or repeated measures (**f**) or ordinary (**e**, **j**, **k**) two-way ANOVA with Sidak’s multiple comparisons test. MFI = mean fluorescence intensity. Source data are provided as a Source Data file.

As previously described, *Ifnar*^*-/-*^ mice exhibit immune defects due to their lack of type I IFN signaling. We sought to confirm that bystander activation occurs independent of genetic IFNAR deficiency using an α-IFNAR-treated C57BL/6 mouse model^19,54^. Despite administration of a higher dose of ZIKV (1.4x10^6^ PFU) compared to the standard dose for *Ifnar*^*-/-*^ mice (4x10^5^ PFU), α-IFNAR-treated C57BL/6 mice exhibited significantly lower ZIKV viremia compared to *Ifnar*^*-/-*^ mice (Fig. 7a). Despite a lower viral load, α-IFNAR-treated C57BL/6 mice showed comparable NKG2D expression on CD8^+^ T cells in the spleen (Fig. 7b–d). Furthermore, CD8^+^ T cells isolated from the spleens of *Ifnar*^*-/-*^ or α-IFNAR-treated ZIKV-infected C57BL/6 mice exhibited similar levels of antigen-independent cytotoxicity against YAC-1 tumor cells, further suggesting that bystander activation and cytotoxic function occurs independent of the amount of infectious viral particles (Fig. 7e). In contrast to *Ifnar*^*-/-*^ mice, α-IFNAR-treated ZIKV-infected C57BL/6 mice did not show significant weight loss or neuropathology, and we were unable to detect any infectious ZIKV particles in the brain (Fig. 7f, g). As IFNAR deficiency in structural cells of the CNS is required for ZIKV infection^18,52,54^, it is likely that no CNS infection occurred due to an inability of the IFNAR blocking antibody to cross the blood-brain-barrier. The absence of infection and inflammatory stimuli to recruit immune cells to the brain was evident upon flow cytometry analysis, where α-IFNAR-treated ZIKV-infected C57BL/6 mice show little lymphocyte or CD8^+^NKG2D^+^ T cell infiltration (Fig. 7h–l). Consistent with these results, ZIKV-infected C57BL/6 + α-IFNAR mice lacked production of chemokines previously shown to promote bystander T cell recruitment to inflamed tissues, such as CXCL9, CXCL10, and RANTES^55,56^ (Supplementary Fig. 6). ZIKV-infected C57BL/6 + α-IFNAR mice also showed significantly lower levels of proinflammatory cytokines in the brain compared to ZIKV-infected *Ifnar*^*-/-*^ mice, indicating that viral infection of the brain is required in this context to induce an inflammatory response and bystander T cell-mediated disease (Supplementary Fig. 6). Overall, these results further suggest that while the amount of virus in the brain is not correlated with pathology, an inflammatory stimulus, such as a viral infection, is required to recruit CD8^+^ T cells to the CNS and initiate bystander damage.

## Discussion

Despite evidence that peripheral viral infections can facilitate development of neurological disease, the contribution of viral replication and antiviral immunity to neuropathology has remained unclear. In a mouse model of ZIKV infection, our results demonstrate that while an inflammatory stimulus, such as viral infection, is necessary to induce neurological disease, the amount of infectious virus is not directly correlated with clinical symptoms. Instead, we find that ZIKV-induced neuropathology is mediated by NK cell-like bystander activated CD8^+^ T cells via NKG2D signaling, independent of the viral load in the CNS. Furthermore, we demonstrate that NKG2D-mediated, antigen-independent killing by bystander activated T cells may promote virus-associated neurological disease.

**Fig. 8:**
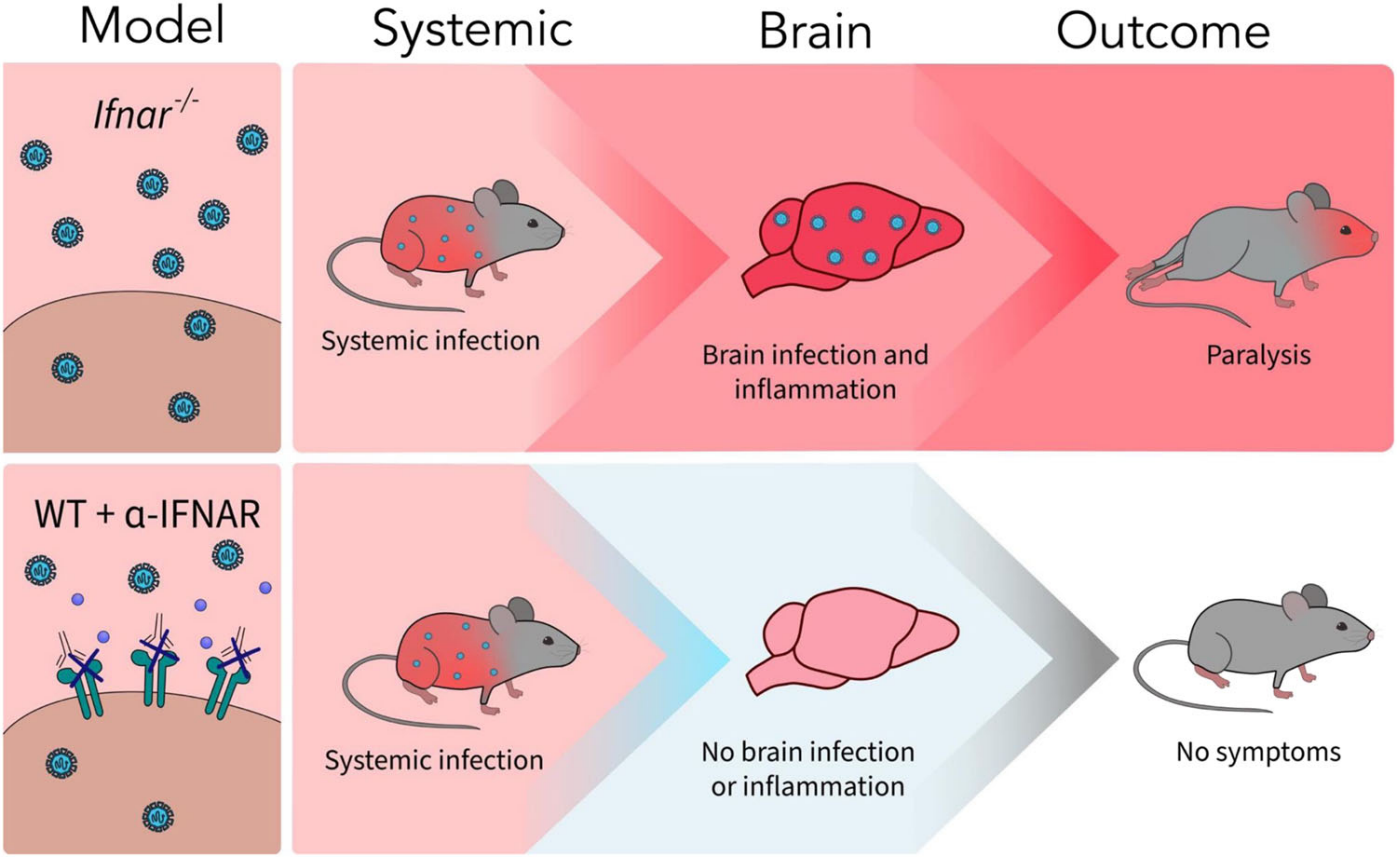
Comparison of ZIKV infection and neurological outcomes in *Ifnar*^*-/-*^ and C57BL/6 + α-IFNAR mouse models. While both *Ifnar*^*-/-*^ and C57BL/6 mice treated with α-IFNAR blocking antibody (WT + α-IFNAR) are susceptible to systemic ZIKV infection, only *Ifnar*^*-/-*^ mice exhibit ZIKV infection and inflammation in the CNS, resulting in neurological symptoms of hindlimb paralysis.

Traditionally, diseases associated with viral infection have been attributed to cytopathic effects of neurotropic viruses. Similarly, the majority of studies of ZIKV infection investigate how ZIKV infection and replication in motor neurons and other CNS cells contribute to pathology^22–25^. In our study, we demonstrate that while viral infection of the CNS is required, the viral load does not drive ZIKV-induced neuropathology. IL-12/18 treatment in vivo significantly reduced viral titers in the serum and brain, despite increased clinical symptoms of paralysis. Therefore, uncontrolled viral replication is not responsible for the clinical symptoms observed following ZIKV infection. Instead, a dysregulated immune response can be attributed to neurological disease seen in this model. CD8^+^ T cells have been previously suggested to contribute to development of ZIKV-induced paralysis, but the mechanism governing this pathogenic immune response remained unclear^18^. Using antibody-mediated CD8 depletion, we confirmed that CD8^+^ T cells play a key role in development of paralysis in *Ifnar*^*-/-*^ mice. In contrast to Jurado et al.^18^, we find that CD8^+^ T cells do not contribute to clearance of infectious viral particles in the brain or periphery, as indicated by similar titers of ZIKV infectious particles in the serum and brains of both PBS and α-CD8 treated, ZIKV-infected mice, despite reduced incidence of neurological symptoms. This difference may be due to our use of a viral plaque assay to quantify infectious ZIKV particles whereas Jurado et al. employed RT-qPCR, where the detection of viral RNA does not necessarily indicate that infectious ZIKV particles are being produced. Using our ZIKV plaque assay, our results confirmed that the infectious viral load in the CNS is not directly associated with ZIKV-induced neurological disease. Additionally, α-IFNAR-treated ZIKV-infected C57BL/6 mice did not develop symptoms of neuropathology, likely due to a lack of CNS infection, inflammation, and CD8^+^NKG2D^+^ T cell infiltration, despite peripheral infection (Fig. 8). This model provides further insight into why many people exhibit mild symptoms following ZIKV infection, while others develop severe neurological disease^13,14^. While the overall viral load does not correlate with bystander activation or neuropathology, our results demonstrate that inflammatory stimuli, such as a viral infection, and the presence of activated immune cells are required to initiate bystander T cell-mediated damage (Fig. 8). Our data further suggest that bystander activated CD8+ T cells are recruited from the periphery by inflammatory stimuli in the brain, rather than the proliferation of brain-resident T cells. α-CD8 antibody depletion effectively depleted CD8+ T cells in the periphery, but is unable to cross the BBB and deplete brain-resident T cells^57,58^. Thus, the lack of bystander activated CD8^+^ T cells in the brain following α-CD8 treatment suggests that these T cells are recruited from the periphery during infection. Further experimentation is needed to confirm these conclusions. In addition, while bystander activated CD8^+^ T cells may contribute to neurological disease in response to inflammatory stimuli without direct infection in the CNS, this hypothesis was not directly tested due to our use of a ZIKV infection model, which does involve infection of the CNS. Nevertheless, we demonstrate evidence of a bystander activated CD8^+^ T cell killing mechanism in virus-induced neuropathology, which can be further investigated in model systems where the virus does not infect the CNS.

The concept of innate-like activation of conventional CD8^+^ T cells, recently named bystander activation, has remained controversial since its inception. Bystander activated T cells have been proposed causes of immunopathology of viral infections but were largely ignored due to their relatively small populations or minimal activation in LCMV or non-fatal mouse hepatitis virus infection^59–62^. More recently, Kim et al. (2018) were the first to describe the pathogenic role of bystander activated T cells in acute hepatitis A (AHA) patients^26^. In this study, they identified bystander activation of non-HAV-specific CD8^+^ T cells and demonstrated their ability to perform antigen-independent killing via NKG2D^26^. While bystander activated CD8^+^ T cells have been identified in liver diseases, our study demonstrates their cytotoxic role in neurological disease. We find that a large proportion of CD8^+^ T cells in the CNS of ZIKV-infected *Ifnar*^*-/-*^ mice show high expression of markers of bystander activated CD8^+^ T cells, such as NKG2D and CXCR6, while remaining CD25^-^, similar to previous studies^26,27,47–49,63^. Despite activation of NK cells via IL-12/18 administration, less than 1% of CD45^+^ cells infiltrating the CNS were NK1.1^+^, indicating that NK cells are not involved in ZIKV-induced neurological disease. Instead, CD8 depletion and NKG2D blockade were sufficient to reduce cell death in the CNS and prevent clinical symptoms of neurological disease, suggesting an NK cell-like mechanism of killing mediated by conventional CD8^+^ T cells. Furthermore, we demonstrate the functional ability of CD8^+^ T cells from ZIKV-infected mice to perform antigen-independent killing against YAC-1 tumor cells, which are known to express NKG2D ligands and are susceptible to NK cell killing^53^. Although the majority of CD8^+^ T cells in the brain were NKG2D^+^, we did observe that a small proportion of CD8^+^ T cells were specific for the immunodominant ZIKV envelope protein epitope. It is possible that these ZIKV-specific CD8^+^ T cells are capable of targeting and killing ZIKV-infected cells via the TCR and could contribute to ZIKV-induced neurological disease. However, since blocking NKG2D significantly reduced cell death and prevented development of paralysis, it is unlikely that antigen-specific T cell killing alone is sufficient to mediate neuropathology.

Despite their resemblance to NK cells, bystander activated T cells pose a significant threat during inflammatory and infectious diseases. CD8^+^ T cells are ‘bystander activated’ in response to hyperinflammation, most notably through IL-15 signaling^26,27,47,64^. Our results suggest that IL-12/18 signaling during viral infection may also contribute to bystander activation, seen through increased CD8^+^ T cell NKG2D expression and antigen-independent cytotoxicity, as well as exacerbated clinical symptoms of paralysis in IL-12/18-treated ZIKV-infected mice. While NK cells possess antigen-independent activation receptors, including NKG2D, they also possess several inhibitory mechanisms to prevent off-target killing of healthy cells. In contrast, bystander activated T cells do not upregulate NK cell inhibitory receptors and are less susceptible to PD-1-mediated inhibition^26,48,55^. Thus, their lack of inhibitory mechanisms renders bystander activated T cells exceptionally dangerous when recruited to sites of viral infection and hyperinflammation, where NKG2D ligand expression has been observed on otherwise healthy cells that are not virally infected^26,31–35^. Without sufficient inhibitory mechanisms that NK cells and antigen-specific T cells possess, bystander activated T cells are licensed to kill any cells expressing stress ligands, resulting in excessive damage. This uncontrolled response may be particularly harmful following recruitment to the CNS, where damage may severely impair essential bodily functions. Further investigation of mechanisms that regulate bystander activation of T cells will be critical to develop effective treatment strategies for bystander activated T cell-mediated diseases.

Although our model involves ZIKV infection of the CNS, this phenomenon may apply to other virus-associated neurological diseases as well. For example, MS has long been suggested to be associated with viral infection, with recent evidence that EBV is the leading cause of the disease^10^. While CD8^+^ T cells have been identified in MS patient brains, and are thought to participate in MS pathogenesis, their role in the disease remains unclear^31,65^. Several studies have also identified NKG2D ligands in the brains of human MS patients and in mouse models of MS, suggesting a potential role for bystander T cells in neurological damage in MS patients^31,66–68^. Similarly, SARS-CoV-2 infection has been associated with neurological disease and long-term symptoms, called long-COVID, but this mechanism of damage remains unclear^6,7^. Analyses of post-mortem brain tissue from COVID-19 patients have demonstrated significant infiltration of CD8^+^ T cells^69,70^. Another study suggested that delayed systemic inflammation and bystander T cell activation was correlated with increased disease severity, while early bystander activation was associated with more mild disease^71^. Further investigation is required to determine if bystander activated CD8^+^ T cells may contribute to COVID-19-associated neuropathology and other virus-induced neurological diseases.

Altogether, we find that viral load is not directly correlated with clinical outcomes of neurological disease. We have identified a mechanism whereby bystander activated CD8^+^ T cells mediate ZIKV-induced neuropathology through antigen-independent killing of stressed cells in the CNS. Furthermore, this mechanism may be broadly applicable to other virus-induced neurological diseases and opens the door for development of effective treatment strategies.

## Methods

### Ethics statement

Mice were used following procedures in accordance with Canadian Council on Animal Care guidelines and approved by the Animal Research Ethics Board at McMaster University (AUP 21-04-12).

### Mice

Breeding pairs of *Ifnar*^−/−^ mice on a C57BL/6 background were provided by Dr. Laurel Lenz (University of Colorado, USA) and then were bred at McMaster’s Central Animal Facility (CAF). C57BL/6 mice were purchased at The Jackson Laboratory (Bar Harbor, ME, USA) and bred at McMaster’s CAF. All mice were housed in McMaster’s Central Animal Facility in specific pathogen-free conditions and temperature controlled environment (21 ± 1 °C) with a 12-h day and night cycle and a maximum of 5 mice per cage. Mice were fed an irradiated Teklad global 18% protein diet (cat# 2918) with ad libitum access to food and water. All experiments were performed using age- and sex-matched 6–18-week-old mice and all findings were repeatable in both sexes.

### ZIKV Infection

The Puerto Rican (PRVABC-59) strain of ZIKV was provided by Dr. David Safronetz (Public Health Agency of Canada, Winnipeg). The virus was propagated in Vero76 cells (ATCC CRL-1587), harvested in DMEM supplemented with 2% FBS, and stored at −80 °C. *Ifnar*^*-/-*^ mice were infected via the footpad by injecting 4 × 10^5^ PFU in 30–50 μL under gas anesthesia. C57BL/6 mice were infected with 50μL ZIKV in both hindlimb footpads to a total of 1.4x10^6^ PFU under gas anesthesia. Endpoint was defined as weight loss >20% or development of hindlimb (or in some rare cases, forelimb) paralysis. Paralysis was measured on a score from 0 to 3, where 0 indicated no apparent neurological symptoms. Hindlimb weakness (1) was identified as mice with altered gait and impaired grip or partial paralysis in one or both hindlimbs. Mice were euthanized at the onset of complete paralysis of one (2) or both (3) hindlimbs.

### In vivo treatments

All intraperitoneal (IP) injections were administered in 200μL of PBS. IP injections of 150ng of recombinant murine IL-12 p70 (210-12, Peprotech) and 750ng of recombinant murine IL-18 (B004-5, Mbl International Corporation) were administered 1- and 2-days post-infection (dpi) or 0-3 dpi. To block NKG2D receptor signaling, 100 μg of anti-mouse α-NKG2D (BioXCell #BE0111 clone: HMG2D) were administered IP at 1, 4, and 6 dpi. CD8^+^ T cells were depleted using 100μg anti-mouse α-CD8 (BioXCell #BE0061 clone: 2.43) at −1 and 0 dpi. To investigate ZIKV infection and bystander activation in mice without genetic deficiency of IFNAR, C57BL/6 mice were administered anti-mouse α-IFNAR (BioXCell #BE0241 clone: MAR1-5A3) at doses of 1mg (−1 dpi) and 0.5mg (0, 1, 3, and 5 dpi).

### Blood and brain cell isolation and flow cytometry

Blood for flow cytometry analysis was collected from the facial vein in tubes containing anticoagulant (BD Biosciences ACD solution A) and red blood cells were lysed by ACK treatment.

Although ZIKV infects both the spinal cord and brain^18,20,22^, brain tissue is commonly used in ZIKV investigation and cell death in the cerebral cortex and cerebellum was previously shown to correlate with clinical symptoms^18,22^. For brain cell isolation, mice were anaesthetized (100 mg/kg ketamine and 10 mg/kg xylazine) and perfused with 20 mL of PBS. Brains were harvested and homogenized on ice using a 2 mL Dounce homogenizer in 1X HBSS. Cells were separated from myelin by density centrifugation using a 70/30 percoll gradient (GE Healthcare Life Sciences).

Isolated cells were stained for viability using Fixable Viability Stain 510 (BD Biosciences #564406) or eFluor™ 780 fixable viability dye (eBioscience #65-0865-14). Before extracellular staining, cells were incubated with anti-mouse CD16/CD32 at 4 °C (eBioscience #14-0161-82). For tetramer analyses, cells were stained for 30 minutes at room temperature with APC-conjugated ZIKV Env_294-302_ tetramer (IGVSNRDFV) provided by the National Institutes of Health Tetramer Core Facility. Extracellular staining was performed in FACs buffer (0.2% BSA in PBS) at 4 °C using the following antibodies in an appropriate combination of fluorophores: Alexa Fluor 700 anti-mouse CD45 (eBioscience #56-0451-82 clone: 30-F11), Pacific Blue anti-mouse CD3 (Biolegend #100214 clone: 17A2), APC anti-mouse CD3 (Biolegend #100236 clone: 17A2), PECF594 anti-mouse CD3 (BD Biosciences #562286 clone: 145-2C11), APC anti-mouse CD8 (Biolegend # 100712 clone: 53-6.7), Alexa Fluor 488 anti-mouse CD8 (Biolegend #100723 clone: 53-6.7), PE-Cy7 anti-mouse CD11b (Biolegend #101216 clone: M1/70), PE anti-mouse CD11b (Biolegend #101208 clone: M1/70), PerCP-Cy5.5 anti-mouse CD11b (Biolegend #101228 clone: M1/70), PE anti-mouse NK1.1 (eBioscience #12-5941-83 clone: PK136), BV421 anti-mouse NK1.1 (Biolegend #108741 clone: PK136), PE anti-mouse NKG2D (eBioscience #16-5882-85 clone: CX5), PE-Dazzle594 anti-mouse CD44 (Biolegend #103056 clone: IM7), Alexa Fluor 488 anti-mouse CD25 (eBioscience #53-0251-82 clone: PC61.5), BV421 anti-mouse CXCR6 (Biolegend # 151109 clone: SA051D1), BV605 anti-mouse TCR β (Biolegend #109241 clone: H57-597). For intracellular staining of IFN-γ, spleen cells were rapidly processed and stained for surface markers, fixed and permeabilized using BD Cytofix/Cytoperm following manufacturer’s instructions (BD Biosciences #554715), and stained with APC anti-mouse IFN-γ (Biolegend #505810 clone: XMG1.2) in BD Perm/Wash as described previously^72,73^. All cells were fixed with 2% PFA and resuspended in FACs buffer before data acquisition on the flow cytometer. All flow cytometry was conducted on a BD LSRFortessa (BD Bioscience), collected using FACSDiva Software (version 8.0, BD Biosciences), and analyzed using FlowJo software (version 10.8.1, BD Biosciences).

### Plaque assay

To detect viremia in ZIKV-infected mice, blood was collected from the facial vein at various days post-infection and serum was isolated by centrifugation. To quantify viral replication in the CNS, brains were isolated as previously described and homogenized using a Bio-Gen PRO200 Homogenizer in a volume of HBSS 2x the brain weight. The samples were centrifuged twice and supernatants were collected. All samples were stored at −80 °C before viral quantification by plaque assay.

Vero76 cells (ATCC CRL-1587) were grown to confluency in 12-well plates in DMEM supplemented with 10% fetal bovine serum (FBS), 2 mM l-glutamine, 10 mM HEPES, 100 U/mL penicillin, and 100 μg/mL streptomycin. Serum and brain supernatant samples were serially diluted in 0% DMEM and incubated with the monolayer for 1 h. The cells were then overlayed with 2 mL of a 1:1 mixture of 2% methylcellulose (Sigma Aldrich, MO, USA) and 2X MEM with Earle’s salts (Gibco; Thermo Fisher Scientific, Waltham, MA, USA) supplemented with 2% FBS and the above additives. Four days later, the overlay was removed, and the cells were fixed and stained with crystal violet. Plaques were quantified using an inverted microscope.

### Spleen cell isolations

Spleens were isolated from euthanized mice and gently homogenized into a single-cell suspension using the plunger of a 1 mL syringe. For flow cytometry, spleen cell suspensions were incubated with ACK lysis buffer to lyse red blood cells and then stained for flow cytometry analysis. For cytotoxicity assays, CD8^+^ T cells from naïve and ZIKV-infected mice were isolated from spleen cell suspensions via negative selection using the EasySep™ Mouse CD8+ T Cell Isolation Kit (STEMCELL Technologies #19853).

### Antigen-independent cytotoxicity assay

Antigen-independent killing was assessed via flow cytometry-based cytotoxicity assay against YAC-1 tumor cells (ATCC TIB-160) cultured in RPMI with 10% FBS and previously described additives. YAC-1 cells were labeled with carboxyfluorescein succinimidyl ester (CFSE; Sigma-Aldrich) then co-cultured with isolated spleen CD8^+^ T cells or bulk brain cells in a U-bottom 96-well culture plate at the indicated effector:target (E:T) ratios for 5 h at 37 °C. 50,000 target cells were used per well and performed in duplicates. After the 5-hour incubation, cells were stained with Fixable Viability Dye eFluor 780 (eBioscience). Cells were gated on CFSE-labeled events and cell death was calculated by gating on the Fixable viability dye eFluor 780-positive gate. Percent specific cell lysis was calculated as: ((% lysis – % basal tumor lysis)/ (100- % basal tumor lysis)) × 100.

### Histology

Brains were isolated as previously described and fixed in 4% paraformaldehyde for 24-48 hours and then stored in 70% ethanol. Tissue was embedded in paraffin and sagittal sections were stained with hematoxylin and eosin stains. Slides were subsequently scanned with Aperia ScanScope XT (Leica Biosystems). For brain lymphocyte quantification via histology, we established a score from 0 to 3, with 0 being no lymphocyte infiltration and 3 being high levels of lymphocyte infiltration and perivascular cuffing in the cerebral cortex. Blinded brain histology scores were assigned by two individuals and the average score for each brain was used for comparison.

Terminal deoxynucleotidyl transferase dUTP nick end labeling (TUNEL) was performed on sagittal sections of paraffin-embedded brains using the ApopTag® Plus In Situ Apoptosis Fluorescein Detection Kit (Sigma Aldrich, #S7111) and counterstained with 4′,6-diamidino-2-phenylindole (DAPI). Images were obtained using the Leica STELLARIS 5 Spectral Confocal System. TUNEL+ nuclei were counted in 3 randomly selected fields from each brain region using ImageJ.

### Cytokine analysis

Serum samples were collected as previously described. IFN-γ was detected in serum samples using the Mouse IFN-γ Duoset ELISA kit (R&D). Brain supernatants were inactivated by 1% FBS 1% Triton X-100 diluted in PBS and subjected to a 44-plex Luminex assay (Eve Technologies).

### Statistical analysis

Statistical analysis was conducted using GraphPad Prism version 9.1.1. Data are presented as mean ± SEM unless otherwise described. Differences were analyzed using a Student’s *t* test (parametric data), one-way ANOVA (if more than two groups were analyzed), or repeated measures or ordinary two-way ANOVA (if more than two groups were analyzed with two independent variables). Post-hoc analysis was performed using Tukey’s multiple comparison test unless otherwise stated.

### Reporting summary

Further information on research design is available in the Nature Portfolio Reporting Summary linked to this article.

### Supplementary information

Supplementary Information

Reporting Summary

### Source data

Source Data

## Data Availability

The main data supporting the findings of this study are available within the article and its Supplementary Information files. Source data are provided with this paper.
