# Supplementary material for: Bystander activated CD8+ T cells mediate neuropathology during viral infection via antigen-independent cytotoxicity
Source: Nat Commun. 2024 Feb 5;15:896. doi: 10.1038/s41467-023-44667-0 (PMC10844499; doi:10.1038/s41467-023-44667-0)
Supplement: Supplementary file 1 — Supplementary Information [file 41467_2023_44667_MOESM1_ESM.pdf]

Supplementary Fig. 1

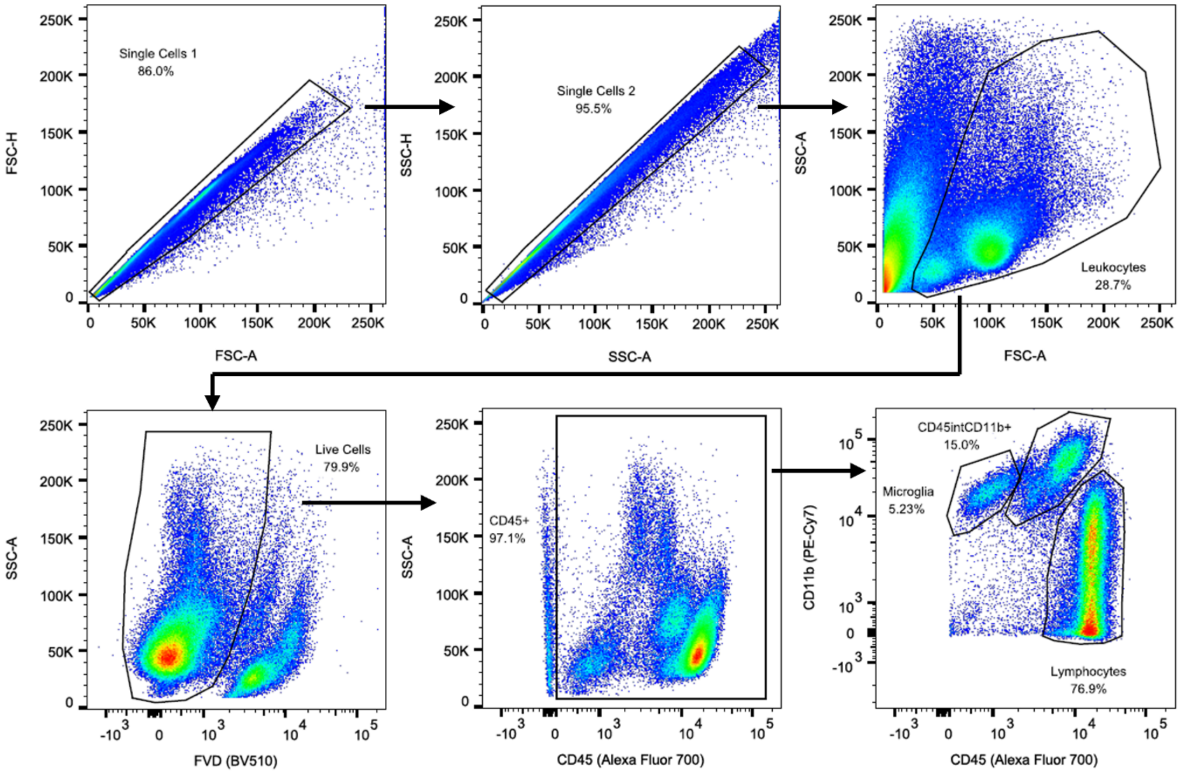

Supplementary Fig. 1. Gating strategy for analysis of brain immune cells following ZIKV infection. Representative flow cytometry plots of brain immune cells. Cells are gated on singlets twice (FSC-A/FSC-H, SSC-A/SSC-H) then gated on leukocytes. Live cells are gated on CD45 and assessed for microglia ($CD45^{lo}CD11b^{+}$), activated macrophages/microglia ($CD45^{int/hi}CD11b^{+}$), and lymphocytes ($CD45^{hi}CD11b^{low/int}$). FVD = fixable viability dye.

Supplementary Fig. 2

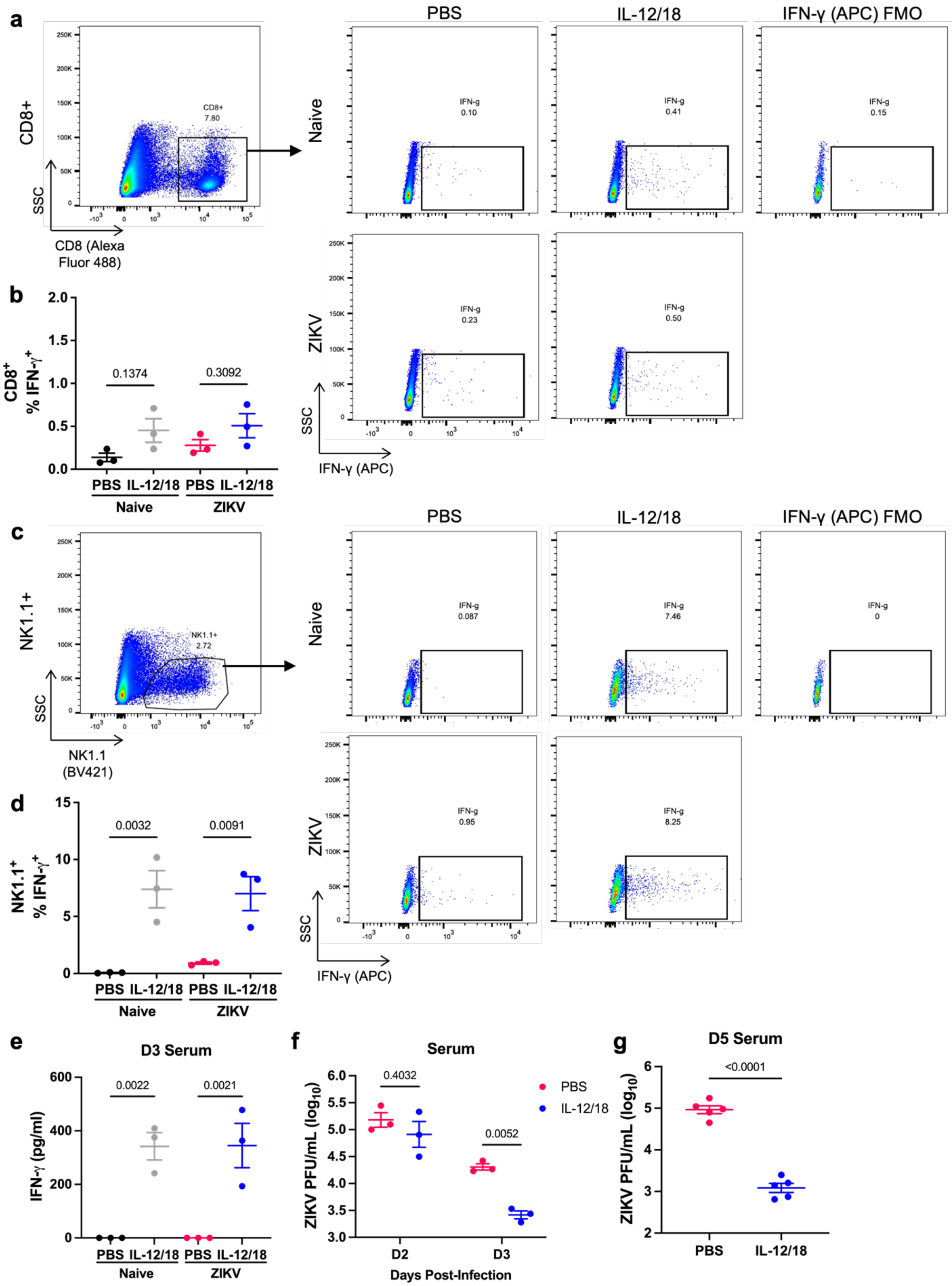

Supplementary Fig. 2. IL-12/18 treatment induces NK cell IFN-γ production. PBS (naïve) and ZIKV-infected mice were administered IP treatments of PBS or IL-12/18 at 1 and 2 dpi. Spleens were harvested and cells isolated for flow cytometry analysis at 3 dpi. A) Representative gating of spleen $CD8^+$ T cells and $CD8^+$ T cell intracellular IFN-γ at 3 dpi. B) Quantification of spleen $CD8^+$ T cell intracellular IFN-γ (n=3). C) Representative gating of spleen $NK1.1^+$ cells and NK cell intracellular IFN-γ at 3 dpi. D) Quantification of spleen NK cell intracellular IFN-γ (n=3). E) Serum was collected at 3 dpi for quantification of IFN-γ by ELISA (n=3). F) Serum was collected from ZIKV-infected mice at 2 and 3 dpi for quantification by plaque assay (n=3). G) Serum was collected from ZIKV-infected mice at 5 dpi for ZIKV plaque assay (n=5). Data represent mean ± SEM (B, D, E-G). Statistical significance was determined by two-way ANOVA with Sidak's multiple comparisons test (B, D-F) or two-tailed student's t-test (G). FMO = fluorescence minus one. Source data are provided as a Source Data file.

Supplementary Fig. 3

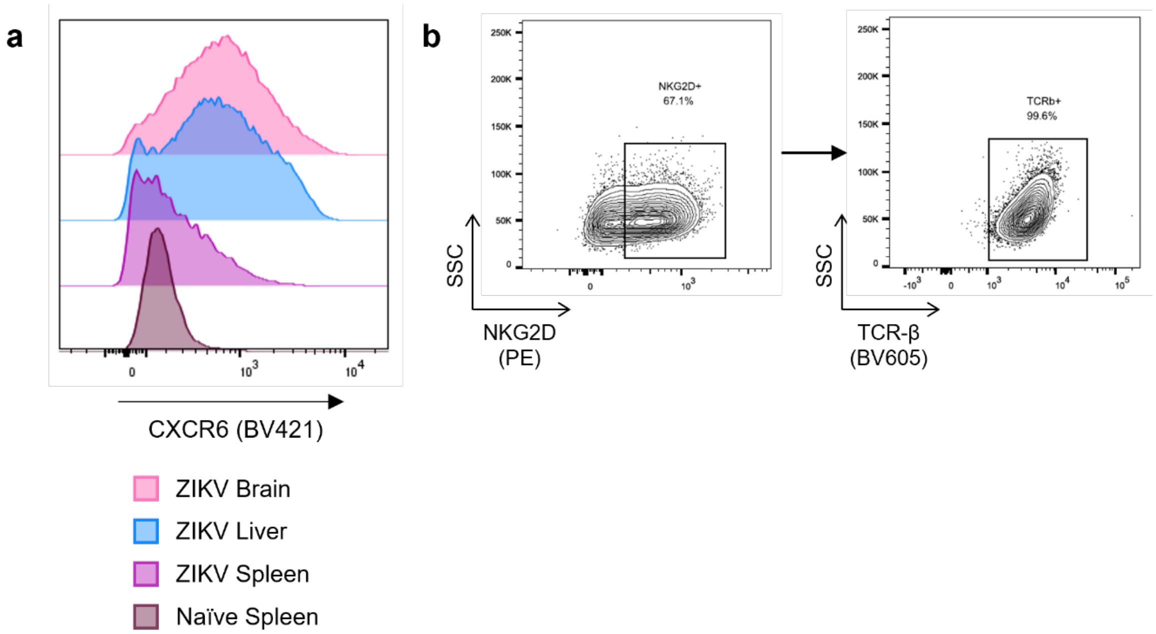

Supplementary Fig. 3. ZIKV infection in mice elicits bystander activation of conventional CD8⁺ T cells. A) Representative histograms of CXCR6 expression by CD8⁺ T cells from various organs of naïve and ZIKV infected mice. B) Representative flow cytometry plot demonstrating TCR-β expression on NKG2D⁺CD8⁺ T cells isolated from ZIKV-infected mouse brains.

Supplementary Fig. 4

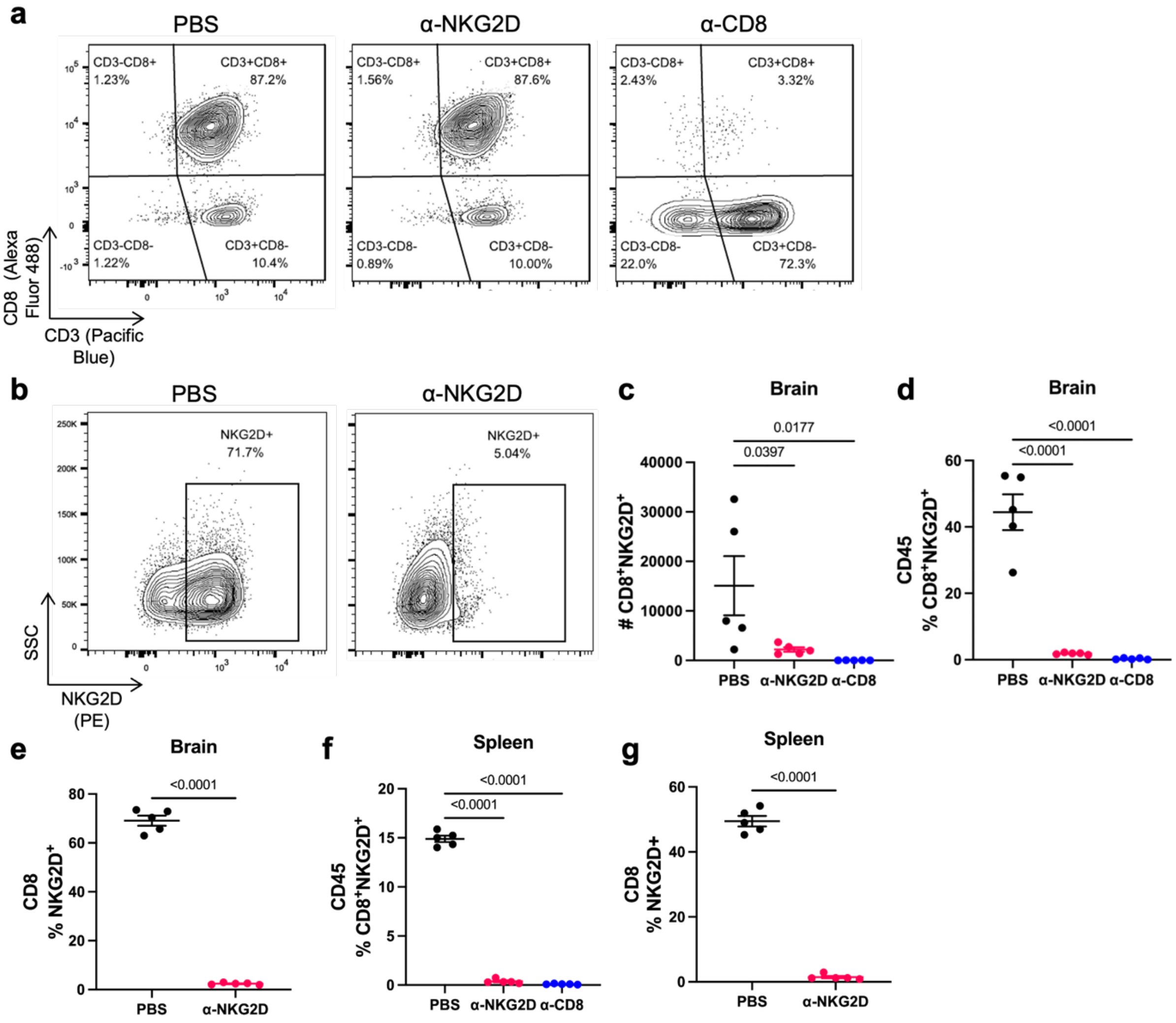

Supplementary Fig. 4. Antibody depletion of CD8 and blockade of NKG2D in ZIKV-infected mice. A) Representative flow cytometry plots of lymphocytes from the brains of PBS, α-NKG2D, and α-CD8-treated ZIKV-infected mice. B) Representative flow cytometry plots of brain $CD8^+$ T cell NKG2D expression following PBS or α-NKG2D treatment of ZIKV-infected mice. C) Count of $CD8^+NKG2D^+$ T cells in the brains of PBS, α-NKG2D, and α-CD8-treated ZIKV-infected mice (n=5). D) Proportion of brain $CD45^+$ cells that were $CD8^+NKG2D^+$ (n=5). E) Proportion of brain $CD8^+$ T cells from ZIKV-infected mice that were $NKG2D^+$ (n=5). F)

Proportion of spleen $CD45^+$ cells that were $CD8^+NKG2D^+$ (n=5). G) Proportion of spleen $CD8^+$ T cells that were $NKG2D^+$ (n=5). Data represent mean ± SEM (C-G). Statistical significance was determined by one-way ANOVA with Dunnett's multiple comparisons test (C, D, F) or two-tailed student's t-test (E, G). Source data are provided as a Source Data file.

Supplementary Fig. 5

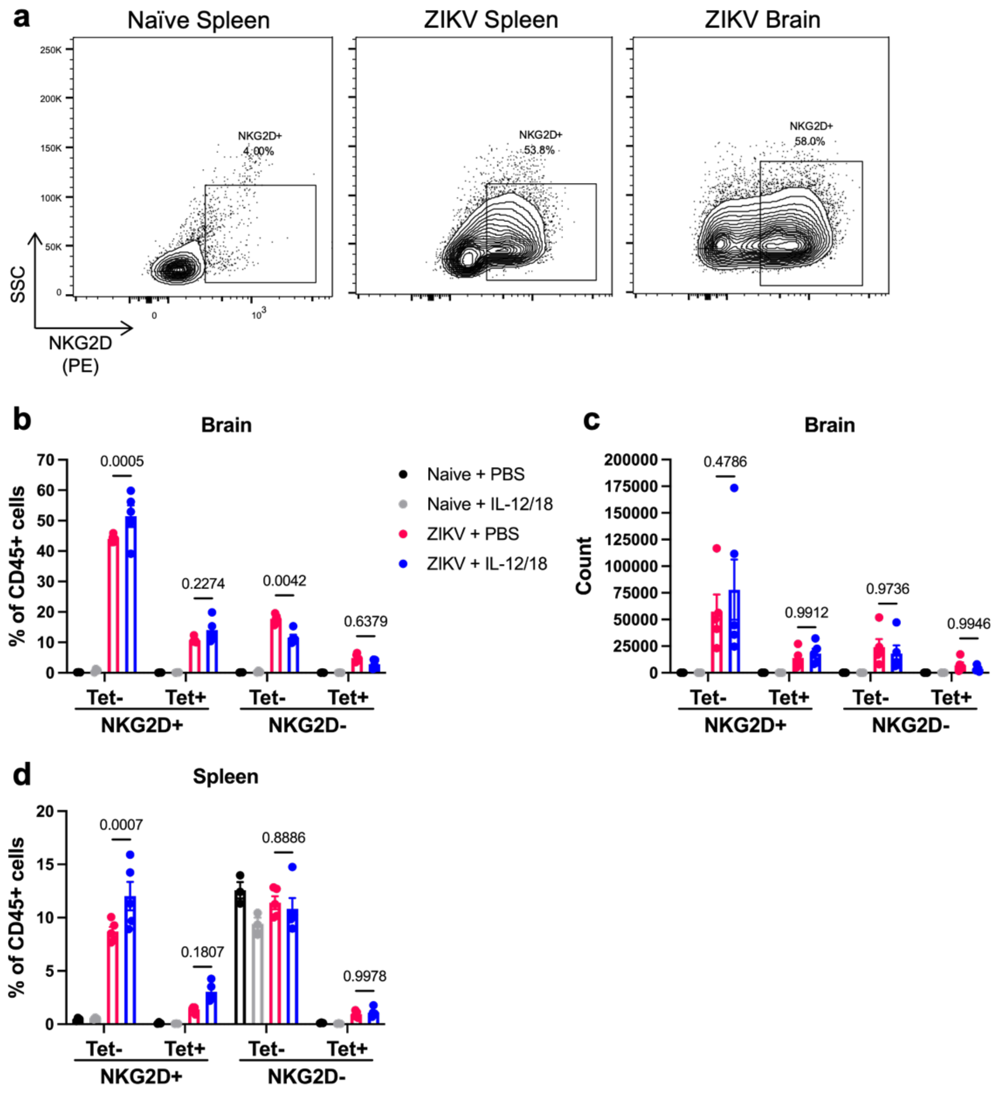

Supplementary Fig. 5. NKG2D expression and tetramer staining on spleen and brain CD8⁺ T cells from naïve or ZIKV-infected mice. A) Representative flow cytometry plots of CD8⁺ T cells from the spleens and brains of naïve (PBS-treated) or ZIKV-infected mice. B-D) *Ifnar-/-* mice were infected with PBS (naïve) or 4x10⁵ PFU ZIKV ZIKV and treated with PBS or IL-12/18 at 1 and 2 dpi. Brains and spleens were harvested for flow cytometry analysis. B) The proportion of brain CD45⁺ cells that were CD8⁺, gated on NKG2D and ZIKV tetramer staining (n=3, 5). C) Counts of CD8⁺ T cells gated on NKG2D and ZIKV tetramer staining (n=3, 5). D)

The proportion of spleen $CD45^+$ cells that were $CD8^+$, gated on NKG2D and ZIKV tetramer staining (n=3, 5). Data represents mean ± SEM (B-D). Statistical significance was determined by two-way ANOVA with Tukey's multiple comparison test (B-D). Tet = Tetramer. Source data are provided as a Source Data file.

Supplementary Fig. 6

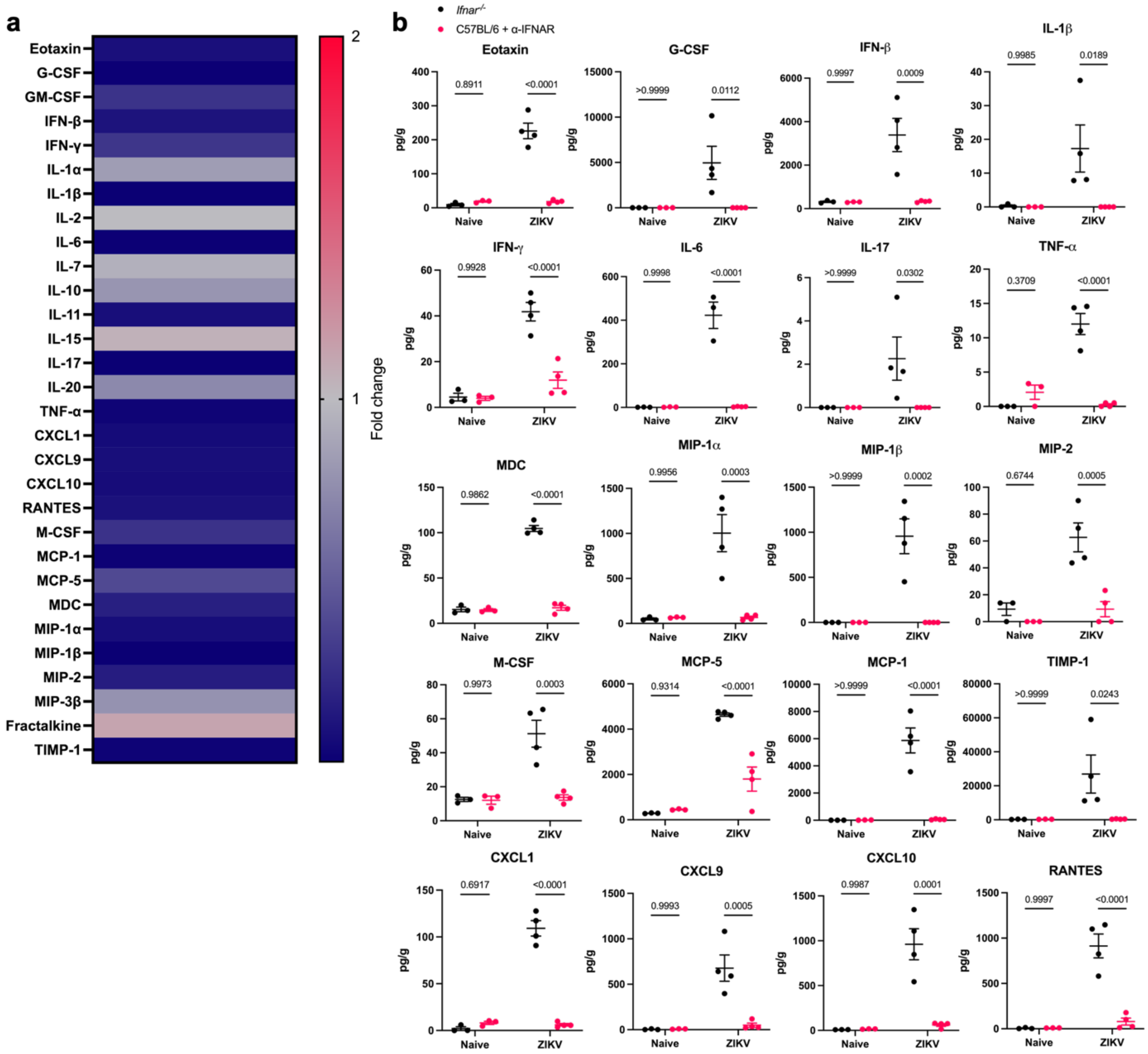

Supplementary Fig 6. α-IFNAR-treated C57BL/6 mice are protected from ZIKV-induced inflammation in the brain. C57BL/6 mice were treated with α-IFNAR antibody at -1, 0, 1, 3, 5 dpi and infected via FTPD with $1.4x10^6$ ZIKV PFU. *Ifnar*$^{-/-}$ mice were infected via FTPD with $4x10^5$ ZIKV PFU. Brains were collected at 7 dpi and homogenized for multiplex cytokine analysis. A) Heatmap depicting fold change of mean cytokine concentrations of brains from ZIKV-infected C57BL/6 + α-IFNAR mice (n=4) compared to *Ifnar*$^{-/-}$ mice (n=4). B) Graphs of cytokines/chemokines of interest that were significantly lower in the brains of C57BL/6 + α-

IFNAR mice compared to *Ifnar*$^{-/-}$ mice (n=3, 3, 4, 4). Data represent mean ± SEM and statistical significance was determined by two-way ANOVA with Sidak's multiple comparisons test. Source data are provided as a Source Data file.
