# Supplementary material for: Bystander activated CD8+ T cells mediate neuropathology during viral infection via antigen-independent cytotoxicity
Source: Nat Commun. 2024 Feb 5;15:896. doi: 10.1038/s41467-023-44667-0 (PMC10844499; doi:10.1038/s41467-023-44667-0)
Supplement: Supplementary file 2 — Reporting Summary [file 41467_2023_44667_MOESM2_ESM.pdf]

nature portfolio

Corresponding author(s): Ali A Ashkar

Last updated by author(s): Dec 6, 2023

Reporting Summary

Nature Portfolio wishes to improve the reproducibility of the work that we publish. This form provides structure for consistency and transparency in reporting. For further information on Nature Portfolio policies, see our Editorial Policies and the Editorial Policy Checklist.

Statistics

For all statistical analyses, confirm that the following items are present in the figure legend, table legend, main text, or Methods section.

n/a	Confirmed	
☐	☒	The exact sample size (*n*) for each experimental group/condition, given as a discrete number and unit of measurement
☐	☒	A statement on whether measurements were taken from distinct samples or whether the same sample was measured repeatedly
☐	☒	The statistical test(s) used AND whether they are one- or two-sided<br>*Only common tests should be described solely by name; describe more complex techniques in the Methods section.*
☐	☒	A description of all covariates tested
☐	☒	A description of any assumptions or corrections, such as tests of normality and adjustment for multiple comparisons
☐	☒	A full description of the statistical parameters including central tendency (e.g. means) or other basic estimates (e.g. regression coefficient) AND variation (e.g. standard deviation) or associated estimates of uncertainty (e.g. confidence intervals)
☐	☒	For null hypothesis testing, the test statistic (e.g. *F*, *t*, *r*) with confidence intervals, effect sizes, degrees of freedom and *P* value noted<br>*Give P values as exact values whenever suitable.*
☒	☐	For Bayesian analysis, information on the choice of priors and Markov chain Monte Carlo settings
☒	☐	For hierarchical and complex designs, identification of the appropriate level for tests and full reporting of outcomes
☐	☒	Estimates of effect sizes (e.g. Cohen's *d*, Pearson's *r*), indicating how they were calculated

Our web collection on statistics for biologists contains articles on many of the points above.

Software and code

Policy information about availability of computer code

Data collection	Flow cytometry data were collected using FACSDiva Software (version 8.0, BD Biosciences). Histology slides were scanned with Aperio ScanScope XT (Leica Biosystems) and images were taken with Aperio ImageScope v12. Images for TUNEL analyses were obtained using the Leica STELLARIS 5 Spectral Confocal System.
Data analysis	Flow cytometry data were analyzed using Flowjo software (version 10.8.1, BD Biosciences). All graphs were made and statistics calculated using Graphpad Prism 9 (version 9.1.1, GraphPad). Images for TUNEL were analyzed using ImageJ.

For manuscripts utilizing custom algorithms or software that are central to the research but not yet described in published literature, software must be made available to editors and reviewers. We strongly encourage code deposition in a community repository (e.g. GitHub). See the Nature Portfolio guidelines for submitting code & software for further information.

Data

Policy information about availability of data

All manuscripts must include a data availability statement. This statement should provide the following information, where applicable:

- Accession codes, unique identifiers, or web links for publicly available datasets
- A description of any restrictions on data availability
- For clinical datasets or third party data, please ensure that the statement adheres to our policy

The main data supporting the findings of this study are available within the article and its Supplementary Information files. Source data are provided with this paper.

Research involving human participants, their data, or biological material

Policy information about studies with human participants or human data. See also policy information about sex, gender (identity/presentation), and sexual orientation and race, ethnicity and racism.

Reporting on sex and gender	Not applicable
Reporting on race, ethnicity, or other socially relevant groupings	Not applicable
Population characteristics	Not applicable
Recruitment	Not applicable
Ethics oversight	Not applicable

Note that full information on the approval of the study protocol must also be provided in the manuscript.

Field-specific reporting

Please select the one below that is the best fit for your research. If you are not sure, read the appropriate sections before making your selection.

[x] Life sciences [ ] Behavioural & social sciences [ ] Ecological, evolutionary & environmental sciences

For a reference copy of the document with all sections, see nature.com/documents/nr-reporting-summary-flat.pdf

Life sciences study design

All studies must disclose on these points even when the disclosure is negative.

Sample size	Experiments were conducted with at least 3-5 mice per group, chosen to ensure that data were reproducible and held biological significance. These sample sizes are standard for the field and have been previously used to demonstrate differences following ZIKV infection (Elong Ngono et al. Cell Host Mic. 2017; Pardy et al. Nat. Comm. 2019). Larger sample sizes were used for assessment of pathology in accordance with previous studies (Jurado et al. Nat. Mic. 2018; Tripathi et al. Plos Path. 2017).
Data exclusions	All ZIKV-infected mice were assessed for ZIKV viremia by viral plaque assay for inclusion in the study.<br>Mice in the anti-NKG2D blocking experiments were excluded if NKG2D was expressed on greater than 15% of CD8+ T cells in the brain.<br>Mice were assessed for CD8 depletion following anti-CD8 treatment and were required to show less than 10% of brain lymphocytes were CD8 +.
Replication	Technical replicates were performed for all mouse studies and are described in the figure legends. For cytotoxicity assays, each datapoint represents technical duplicates within each biological replicate.
Randomization	Mice were randomly assigned to indicated groups.
Blinding	For brain histology scores, two individuals were blinded to the treatment group and assigned scores independently.<br>Researchers were blinded to treatment group when assessing clinical symptoms of paralysis, and this score was verified by a second individual. The scoring system was established to reflect objective assessment criteria.<br>Flow cytometry data were collected using automated software that did not require intervention by the experimenters. Blinding was not necessary for flow cytometry gating (such as for NKG2D staining) as these were set on negative (uninfected or FMO) controls.

Reporting for specific materials, systems and methods

We require information from authors about some types of materials, experimental systems and methods used in many studies. Here, indicate whether each material, system or method listed is relevant to your study. If you are not sure if a list item applies to your research, read the appropriate section before selecting a response.

Materials & experimental systems

n/a	Involved in the study
	☒ Antibodies
	☒ Eukaryotic cell lines
☒	Palaeontology and archaeology
	☒ Animals and other organisms
☒	Clinical data
☒	Dual use research of concern
☒	Plants

Methods

n/a	Involved in the study
☒	ChIP-seq
	☒ Flow cytometry
☒	MRI-based neuroimaging

Antibodies

Antibodies used

Fc blocking was performed using anti-mouse CD16/CD32 (eBioscience #14-0161-82).
The following antibodies were used in an appropriate combination of fluorochromes:
Alexa Fluor 700 anti-mouse CD45 (eBioscience #56-0451-82 clone: 30-F11)
Pacific Blue anti-mouse CD3 (Biolegend #100214 clone: 17A2)
APC anti-mouse CD3 (Biolegend #100236 clone: 17A2)
PECF594 anti-mouse CD3 (BD Biosciences #562286 clone: 145-2C11)
APC anti-mouse CD8 (Biolegend # 100712 clone: 53-6.7)
Alexa Fluor 488 anti-mouse CD8 (Biolegend #100723 clone: 53-6.7)
PE-Cy7 anti-mouse CD11b (Biolegend #101216 clone: M1/70)
PE anti-mouse CD11b (Biolegend #101208 clone: M1/70)
PerCP-Cy5.5 anti-mouse CD11b (Biolegend #101228 clone: M1/70)
PE anti-mouse NK1.1 (eBioscience #12-5941-83 clone: PK136)
BV421 anti-mouse NK1.1 (Biolegend #108741 clone: PK136)
PE anti-mouse NKG2D (eBioscience #16-5882-85 clone: CX5)
PE-Dazzle594 anti-mouse CD44 (Biolegend #103056 clone: IM7)
Alexa Fluor 488 anti-mouse CD25 (eBioscience #53-0251-82 clone: PC61.5)
BV421 anti-mouse CXCR6 (Biolegend # 151109 clone: SA051D1)
BV605 anti-mouse TCR β (Biolegend #109241 clone: H57-597)
APC anti-mouse IFN-γ (Biolegend #505810 clone: XMG1.2)

Live/dead staining was performed using:
Fixable Viability Stain 510 (BD Biosciences #564406)
eBioscience™ Fixable Viability Dye eFluor™ 780 (eBioscience #65-0865-14)

The following were used for in vivo blockade or depletion:
α-IFNAR (BioXCell #BE0241 clone: MAR1-5A3)
α-NKG2D (BioXCell #BE0111 clone: HMG2D)
α-CD8 (BioXCell #BE0061 clone: 2.43)

Validation

All antibodies are commercially available and were validated by the manufacturers from which they were purchased.

For BioLegend antibodies, validation includes (from https://www.biolegend.com/en-us/quality-control): 1) Staining of 1-3 target cell types with either single- or multi-color analysis detailed in the QC specification (including positive and negative controls). The tested cells can be primary cells and/or cell lines known to be positive or negative for the target antigen. 2) Each batch product is validated by QC testing with a series of dilutions to make sure the product is working within expected antibody titer range. 3) Each batch is compared to an internally established "gold standard" to maintain batch-to-batch consistency.

For eBioscience/Invitrogen antibodies, validation includes (from https://www.thermofisher.com/ca/en/home/life-science/antibodies/invitrogen-antibody-validation.html#2-part-testing-flyer):
Invitrogen™ antibodies are currently undergoing a rigorous 2-part testing approach.
Part 1. Target specificity verification.
Helps ensure the antibody will bind to the
correct target; our antibodies are being tested
using at least one of the following methods:
Knockout, Knockdown, Independent antibody verification, Cell treatment, Relative expression, Neutralization, Peptide array, SNAP-ChIP™ validation, Immunoprecipitation/mass spectrometry

Part 2. Functional application validation.
These tests help ensure the antibody works in particular application(s) of interest, which may include (but are not limited to):
Western blotting, Immunofluorescence imaging, Flow cytometry, ChIP, Immunohistochemistry

All flow cytometry and in vivo antibody performance was validated using controls (eg. spleen vs brain, noninfected vs infected mice,

cytokine treatment), titration, and fluorescence minus one (FMO) samples.

Eukaryotic cell lines

Policy information about cell lines and Sex and Gender in Research

Cell line source(s)	Vero76 cells (ATCC CRL-1587) and YAC-1 cells (ATCC TIB-160) were purchased from ATCC.
Authentication	All cell lines used were authenticated by the manufacturers and checked morphologically under the microscope.
Mycoplasma contamination	The cell line was not tested for mycoplasma contamination.
Commonly misidentified lines (See ICLAC register)	No commonly misidentified cell lines were used.

Animals and other research organisms

Policy information about studies involving animals; ARRIVE guidelines recommended for reporting animal research, and Sex and Gender in Research

Laboratory animals	6-18 week old Ifnar-/- mice on a C57BL/6 background and C57BL/6 mice of both sexes were used. All experiments used age-matched and sex-matched mice. All mice were housed in McMaster's Central Animal Facility in specific pathogen-free conditions and temperature controlled environment (21 degrees Celsius) with a 12-hour day and night cycle and a maximum of 5 mice per cage. Mice were fed an irradiated Teklad global 18% protein diet (cat# 2918) with ad libitum access to food and water.
Wild animals	The study did not involve wild animals.
Reporting on sex	All experiments used sex-matched mice and all findings were repeatable in both sexes.
Field-collected samples	The study did not involve samples collected from the field.
Ethics oversight	Mice were used following procedures approved by the McMaster University Animal Research Ethics Board (AUP 21-04-12) and in accordance with the Canadian Council on Animal Care guidelines.

Note that full information on the approval of the study protocol must also be provided in the manuscript.

Plants

Seed stocks	Not applicable
Novel plant genotypes	Not applicable
Authentication	Not applicable

Flow Cytometry

Plots

Confirm that:

- [x] The axis labels state the marker and fluorochrome used (e.g. CD4-FITC).
- [x] The axis scales are clearly visible. Include numbers along axes only for bottom left plot of group (a 'group' is an analysis of identical markers).
- [x] All plots are contour plots with outliers or pseudocolor plots.
- [x] A numerical value for number of cells or percentage (with statistics) is provided.

Methodology

Sample preparation	Blood for flow cytometry analysis was collected from the facial vein in tubes containing anticoagulant (BD Biosciences ACD solution A) and red blood cells were lysed by ACK treatment.<br>For brain cell isolation, mice were anaesthetized (100 mg/kg ketamine and 10 mg/kg xylazine) and perfused with 20mL of PBS. Brains were harvested and homogenized on ice using a 2 mL Dounce homogenizer in 1X HBSS. Cells were separated from myelin by density centrifugation using a 70/30 percoll gradient (GE Healthcare Life Sciences).

Spleens were isolated from euthanized mice and gently homogenized into a single cell suspension using the plunger of a 1 mL syringe. For flow cytometry, spleen cell suspensions were incubated with ACK lysis buffer to lyse red blood cells and then stained for flow cytometry analysis.

Isolated cells were stained for viability using Fixable Viability Stain 510 (BD Biosciences #564406) or eFluor™ 780 fixable viability dye (eBioscience #65-0865-14). Before extracellular staining, cells were incubated with anti-mouse CD16/CD32 at 4 degrees Celsius (eBioscience #14-0161-82). For tetramer analyses, isolated cells were stained for 30 minutes at room temperature with APC-conjugated ZIKV Env294-302 tetramer (IGVSNRDFV) provided by the National Institutes of Health Core Tetramer Core Facility. Extracellular staining was performed in FACs buffer (0.2% BSA in PBS) at 4 degrees Celsius.

For intracellular staining, cells were fixed and permeabilized using BD Cytofix/Cytoperm following manufacturer's instructions (BD Biosciences #554715) and stained with APC anti-mouse IFN-γ (Biolegend #505810 clone: XMG1.2) in BD Perm/Wash. All cells were fixed with 2% PFA and resuspended in FACs buffer before data acquisition on the flow cytometer. All flow cytometry was conducted on a BD LSRFortessa (BD Bioscience) and analyzed using FlowJo v10 software.

Instructment

Instrument: BD LSRFortessa Cell Analyzer

Software: Data was collected using FACSDiva software (version 8.0, BD Biosciences) and analyzed using Flowjo (BD Biosciences).

Cell population abundance: No cell sorting was used in this study.

Gating strategy: Samples were first gated on singlets from FSC-A and FSC-A, then SSC-A and SSC-H (where events with high FSC-A but dissimilarly high FSC-H, or SSC-A vs SSC-H, were excluded). From singlets, we gated on a leukocyte gate which excludes low FSC and low to high SSC (debris, red blood cells). Leukocyte gate typically included cells with FSC >30000 and SSC >10000. Next, we gated live events, which were low for amine-reactive fixable viability dye. We next gated CD45+ cells. For the brain analysis, brains were gated based on CD45 and CD11b expression to determine microglia (CD45loCD11b+), activated microglia/macrophages (CD45hiCD11b+), or lymphocytes (CD45hiCD11b lo/int). Representative gating is shown in Figure S1. Subsequent gating for CD3, CD8, or NK1.1 was performed on lymphocyte gate (or from CD45 gate for spleen or blood). From CD8 gate, NKG2D, CXCR6, TCRbeta, CD25, IFN-gamma, or ZIKV tetramer gates were applied (representative gating shown throughout figures). Boundaries between positive and negative populations were determined by on the separate in the FACs plot, Fluorescence Minus One (FMO) controls where appropriate, or compared to noninfected controls (eg. NKG2D gate set based on naive spleen or blood CD8+ T cells that are established in the literature to be largely NKG2D-). Furthermore, all gates were also compared to previously published analyses.

☒ Tick this box to confirm that a figure exemplifying the gating strategy is provided in the Supplementary Information.
